# Recent Advances in Nanomaterials for Diagnosis, Treatments, and Neurorestoration in Ischemic Stroke

**DOI:** 10.3389/fncel.2022.885190

**Published:** 2022-06-28

**Authors:** Xinru Lin, Na Li, Hongli Tang

**Affiliations:** ^1^Department of Anesthesiology, Wenzhou Key Laboratory of Perioperative Medicine, The First Affiliated Hospital of Wenzhou Medical University, Wenzhou, China; ^2^Oujiang Laboratory, Wenzhou Institute, University of Chinese Academy of Sciences, Wenzhou, China

**Keywords:** ischemic stroke, nanomaterials, diagnosis, thrombolytic therapy, neuroprotection

## Abstract

Stroke is a major public health issue, corresponding to the second cause of mortality and the first cause of severe disability. Ischemic stroke is the most common type of stroke, accounting for 87% of all strokes, where early detection and clinical intervention are well known to decrease its morbidity and mortality. However, the diagnosis of ischemic stroke has been limited to the late stages, and its therapeutic window is too narrow to provide rational and effective treatment. In addition, clinical thrombolytics suffer from a short half-life, inactivation, allergic reactions, and non-specific tissue targeting. Another problem is the limited ability of current neuroprotective agents to promote recovery of the ischemic brain tissue after stroke, which contributes to the progressive and irreversible nature of ischemic stroke and also the severity of the outcome. Fortunately, because of biomaterials’ inherent biochemical and biophysical properties, including biocompatibility, biodegradability, renewability, nontoxicity, long blood circulation time, and targeting ability. Utilization of them has been pursued as an innovative and promising strategy to tackle these challenges. In this review, special emphasis will be placed on the recent advances in the study of nanomaterials for the diagnosis and therapy of ischemic stroke. Meanwhile, nanomaterials provide much promise for neural tissue salvage and regeneration in brain ischemia, which is also highlighted.

## Introduction

Stroke is becoming a leading public health problem in the world with an aging population and prolonging life expectancy. which causes 9% of all deaths around the world and is the second leading cause of death after ischemic heart disease (Murray and Lopez, 1997). According to the most recent report of the American Heart Association, the global prevalence of stroke was estimated at 42.4 million in 2015. Among those, ischemic stroke is the most common type, and patients with it account for 87% of stroke patients. Furthermore, direct and indirect medical costs with stroke accounted for 40.1 billion dollars between 2013 and 2014 (Benjamin et al., 2018).

**Figure 1:**
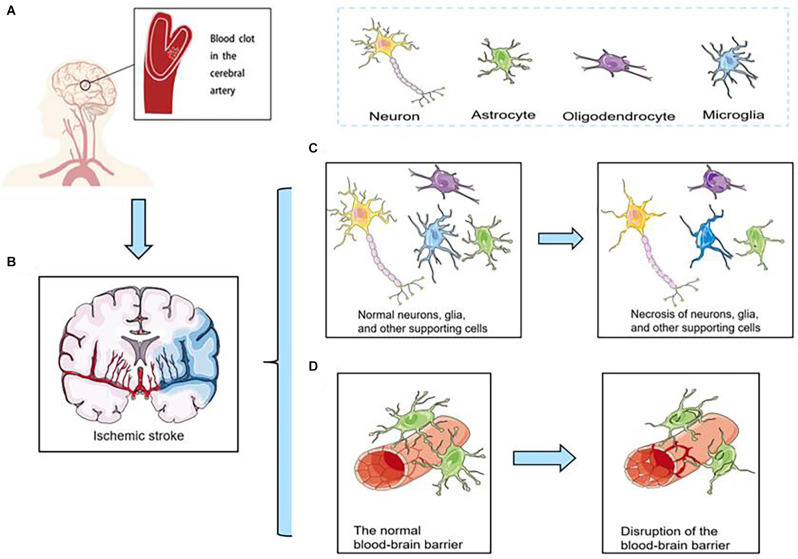
**(A)** Ischemic stroke is caused by abrupt blood vessel occlusion. **(B)** Ischemic stroke initiates a cascade of secondary injury mechanisms in hypoperfused tissue. **(C)** Necrosis of neurons, glia, and other supporting cells. **(D)** Disruption of the blood-brain barrier (BBB).

Although the incidence of stroke has decreased in most regions, it has increased in East Asia, especially in China (Zhao et al., 2008; Johnson et al., 2019). Hence, effectively treating ischemic stroke is important. However, the diagnosis of thrombosis is limited to late stages, and the narrow therapeutic window prevents it from providing reasonable and effective treatment. Therefore, early diagnosis of thrombosis is equally urgent. Ischemic stroke is caused by abrupt blood vessel occlusion (Figure 1A). Which causes insufficient perfusion of oxygenated blood and a limited supply of nutrients to the brain (Cozene et al., 2020). The hypoxic/ischemic condition initiates a cascade of secondary injury mechanisms in hypoperfused tissue (Figure 1B), including necrosis of neurons, glia, and other supporting cells (Figure 1C), and disruption of the blood-brain barrier (BBB; Figure 1D), which in turn causes impairment of brain functions and severe neurological disabilities (Brouns and De Deyn, 2009).

Considering the multitude of pathways involved in tissue damage, the clinical management of stroke addresses strategies that primarily involve early intravenous thrombolysis, mechanical thrombectomy, neuroprotection, and neurorestorative therapies (Prabhakaran et al., 2015; Chamorro et al., 2016; Venkat et al., 2018). With the help of these methods, it is possible to remove blood clots or improve the prognosis of stroke patients. However, drugs used for pharmacological thrombolytic therapy suffer from short half-lives, allergic reactions, or inactivation. Also, these drugs have poor therapeutic efficacy due to their low cumulative efficiency and poor targeting. In addition, mechanical thrombolysis is highly technical and difficult to perform in general hospitals. Moreover, neuroprotective therapy for ischemic stroke is still at an early stage due to the presence of the blood-brain barrier.

Because of the limitations of the above diagnoses and treatment methods, the pursuit of innovative solutions for the management of ischemic stroke remains an open field of research, defining nanomedicine as the biomedical application of nanoscale materials for the diagnosis and therapy of disease (Shi et al., 2010). Nanoparticles mainly include liposomes, polymers, inorganic materials, and proteins (Matsumoto et al., 2019). Due to their unique characteristics, including their large specific surface area, multifunctionality, structural diversity, low immunogenicity, and long circulation time in the blood (Jain, 2009; Zhang et al., 2012; Nitta and Numata, 2013). Therefore, nanoparticles have witnessed considerable achievements in many fields, including medicine, pharmacy, chemical/biological detection, and optics (Lee and Mooney, 2012; Liu et al., 2017). Particularly, in the application of nanotechnology in medicine, nanomedicines are emerging as a promising strategy to improve both the efficacy and safety of thrombolytic therapy.

## Nanomaterials-Based Diagnosis

### Deficiencies of Existing Diagnostic Imaging Techniques

Ischemic stroke has a very limited time window of 4.5–6 h after stroke (Brenna et al., 2020). Therefore, a specific diagnosis of thrombosis is urgent. The most important service that imaging provides to patients with ischemic stroke is to rapidly identify those patients who are most likely to benefit from immediate treatment. Conventional imaging techniques used to diagnose a stroke include computerized tomography (CT), magnetic resonance imaging (MRI), positron emission tomography (PET), and Doppler ultrasonography. But the most widely used stroke diagnostic tools are CT and MRI. Nevertheless, these techniques have unavoidable limitations (Sarmah et al., 2017, 2021; Bonnard et al., 2019; Campbell et al., 2019).

Such as, non-contrast CT of the brain has a high sensitivity for the detection of hemorrhage. But the loss of gray matter–white matter differentiation is a limitation for non-contrast CT to make a positive diagnosis of stroke based on early ischemic changes (Bal et al., 2015). In addition to the non-contrast CT brain scan, diagnosis for stroke includes computed tomography (CT) perfusion scan and CT angiography as routine (Campbell, 2019). Here’s how these two technologies work: based on the intravenous injection of an iodinated contrast agent, *via* a static acquisition to assess the cerebral vasculature is CT angiography, *via* a time-resolved series is CT perfusion. CT angiography is a highly accurate modality for the detection of arterial stenosis and occlusion (Koelemay et al., 2004). In addition, it can also be used to assess the functional extent and direction of collateral flow (Choi et al., 2014), which provides additional prognostic information about the likely extent of tissue injury. CT perfusion is much better for radiological diagnosis of cerebral ischemia than non-contrast CT or CT angiography (Baron et al., 2013). Moreover, CT perfusion can predict patient outcomes more accurately because it has the differentiation of the penumbra from the ischemic core in patients with acute ischemic stroke (Lin et al., 2016; Bivard et al., 2021). However, iodine contrast agents are required for CTA and CTP, which can pose a risk of acute kidney injury (Tsai et al., 2014; McCullough et al., 2016). In addition, both iodinated contrast agents may be associated with some adverse effects in patients, such as hives, nausea, and vomiting (Davenport et al., 2009; Hunt et al., 2009). In addition, when an iodine contrast agent is administered intravenously, inadequate IV access, such as a distal/hand IV or small IV bore, can also contribute to an insufficient bolus profile (Vagal et al., 2019). All these factors limit the wide application of CTA and CTP.

MRI has inherent imaging advantages over CT (Provost et al., 2019). MRI provides a variety of sequences to evaluate the structural and functional characteristics of different brain tissues, including diffusion imaging, perfusion imaging, etc. Diffusion-weighted imaging (DWI) captures changes in water diffusion due to cerebral ischemia and is the most sensitive imaging test for acute cerebral ischemia (Alegiani et al., 2017). And perfusion MRI is similar to CT perfusion in that intravenous gadolinium contrast is tracked through the cerebral circulation, and when contrast is injected into brain tissue, perfusion of normal brain tissue shows a sharp drop in the original signal, which is restored as the contrast is diluted. Hypoperfused brain tissue will have a delayed, scattered, and/or reduced time series of signal intensity compared to healthy tissue (Calamante et al., 2006). Diffusion-and perfusion-weighted imaging has a sensitivity of 97.5% for the diagnosis of acute ischemic stroke (Simonsen et al., 2015). However, MRI also has disadvantages, such as expensive equipment and examinations, and is currently second only to PET in cost. At the same time, MRI examinations are long, and scans are slow. The head scan takes about half an hour. It is also very sensitive to the patient’s body movements and is prone to artifacts (Kim et al., 2019).

The development of the above diagnostic methods is still in its early stages. Besides, the clinical outcomes suggest these methods are not ready for the challenges associated with the diagnosis of ischemic stroke, such as early detection, specific binding, sharp contrast, and continuous monitoring of therapeutic interventions. In addition to this, the contrast agents required for these imaging methods are difficult to pass through various barrier structures, particularly the blood-brain barrier (BBB; Zhang X. et al., 2021). Due to various limitations of current imaging methods for ischemic stroke, optimization of conventional imaging is imminent. Given the excellent properties of nanomaterials, they have been extensively investigated for real-time monitoring of thrombus progression, identification of ischemic semi-dark zones, monitoring blood-brain barrier permeability, monitoring of collateral vessel formation in ischemic areas, and inflammatory progression, etc. (Hoffmann et al., 2018).

### Advantages of Nanomaterials in Diagnostic Imaging

#### Early Identification and Real-Time Monitoring of Thrombus

**Figure 2:**
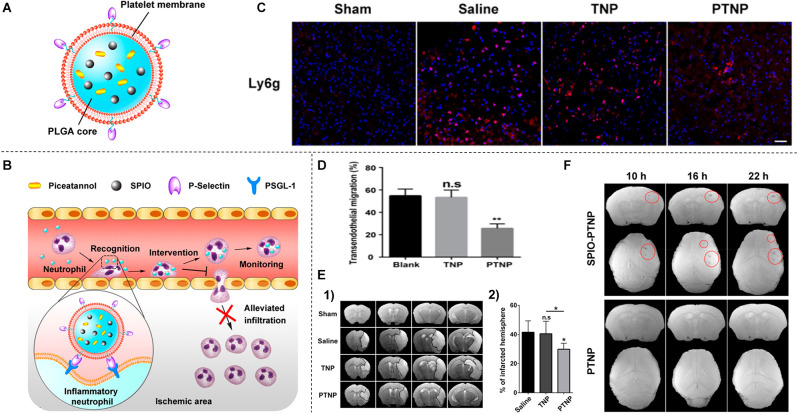
**(A)** Schematic diagram of PTNPs-based multifunctional nanoparticles. **(B)** Selective recognition of inflammatory neutrophils by PTNPs and the subsequent reduction of neutrophil infiltration. **(C)** CLSM images of neutrophil infiltration in the ischemic brain region after treatment with saline, TNPs, and PTNPs. **(D)** Percentage of neutrophils to pre-existing neutrophils after intervention with TNPs or PTNPs. **(E)** T2-weighted images of the ischemic brain at 24 h after treatment with saline, TNPs, and PTNPs hou. The black curve indicates the infarcted area **(1)** and quantitative results of infarct volume **(2)**. **(F)** T2*-weighted coronal and cross-sectional scan images at different time points after injection of SPIO-PTNPs and PTNPs. *P < 0.05; **P < 0.01; n.s means no statistically significant difference. Reprinted (adapted) with permission from Tang et al. (2019). Copyright 2019 American Chemical Society.

Current diagnosis methods based on symptoms of ischemia are not very specific. There are very few positive signs within 6–24 h after the onset of ischemic stroke (Fiebach et al., 2002). As with early diagnosis of ischemic stroke, dynamic monitoring of stroke severity is clinically important, and real-time thrombus imaging allows clinicians to visually identify thrombus burden, distribution, and characteristics to advance personalized thrombolytic therapy (Xia et al., 2017; Tolhuisen et al., 2021). Nanomedicines are being pursued to obtain precise detection of thrombolysis and recanalization in the brain tissue after ischemic stroke. The first type is that the nanoparticles bind to the thrombus site and are specifically labeled by the particles, thus allowing observation of thrombolysis by imaging in the early stages of thrombosis. Neutrophils are an important component of the thrombogenic process, and good thrombus targeting can be achieved by adhering to neutrophils, Tang et al. (2019) synthesized platelet-mimetic nanoparticles (PTNPs; Figure 2A), PTNPs combined with the selective spleen tyrosine kinase inhibitor (piceatannol) and the T2 contrast agent superparamagnetic iron oxide (SPIO) can successfully identify adherent neutrophils through the platelet membrane coating (Figure 2B). In this way, the loaded piceatannol can be delivered to the adherent neutrophils to detach them into circulation, thereby reducing neutrophil infiltration (Figures 2C,D) and infarct size (Figure 2E). In addition, when combined with magnetic resonance imaging, endogenous SPIO can be used to monitor inflammatory neutrophils in real-time and correlate with treatment efficacy (Figure 2F; Tang et al., 2019).

Ultrasound has become the most commonly used imaging method because of its low price and ease of use, but it lacks specificity for the diagnosis of ischemic stroke. Jin et al. (2021) and Li et al. (2018) achieved an accurate diagnosis of ischemic areas by ultrasound through nanomaterials. Jin et al. (2021) applied Xe-encapsulated lipid nanobubbles (Xe-NBs) in the ischemic stroke mouse model. Through timely ultrasound imaging, they found the accumulation of Xe-NBs in the ischemic lesion endows ultrasound contrast imaging with the lesion area in the early stage (Jin et al., 2021). Li et al. (2018) prepared platelet (PLT)-membrane derived bionanobubbles (PNBs) for timely perfusion intervention and ultrasound imaging in acute ischemic stroke. The natural lipid and protein components isolated from PLT membranes gave the PNBs the ability to accurately target lesions. As the accumulation of clusters of PNBs within the lesion increases, intra-lesion PNBs can be monitored by real-time ultrasonography to indicate the severity and dynamic progression of stroke (Li et al., 2018).

In addition, nanoparticles can also optimize the diagnosis of microcomputed tomography (Micro CT). Kim et al. (2013) designed ethylene glycol chitosan (GC) gold nanoparticles (AuNPs). The nanoparticles accumulated in the thrombus could show the presence and extent of primary and recurrent thrombus by computed tomography. Due to their long circulating half-life, GC-AuNPs can be embedded in the fibrin matrix for longer periods of time (up to 3 weeks), allowing for repeated or continuous monitoring of thrombus formation and thrombolysis (Kim et al., 2013).

#### Specific Display of the Ischemic Semi-dark Band

Ischemic stroke produces a core of irreversibly damaged tissue surrounded by a salvageable area called the ischemic penumbra, namely metabolically affected but still viable brain tissue. Such tissue has a high risk of infarction under continuing conditions of reduced CBF but can be rescued through timely intervention (Leigh et al., 2018; Gonzalez-Nieto et al., 2020).

Therefore, there is a great need for methods to create a precise, accurate, and space-time resolution detection for investigating the changes in the ischemic penumbra. Landowski et al. (2020) found HSP72 to be a suitable biomarker of peri-infarct tissue in the ischemic brain (Brea et al., 2015). Therefore, the ischemic penumbra can specifically be identified with nanoparticles such as liposomes loaded with gadolinium and labeled with anti-HSP-72 antibodies (Landowski et al., 2020).

#### Permeability of the Blood-Brain Barrier Can Be Monitored

The blood-brain barrier (BBB) plays a vital role in regulating the trafficking of fluid, solutes, and cells at the blood-brain interface and maintaining the homeostatic microenvironment of the CNS. Under certain neuropathological conditions, such as ischemic stroke, the BBB is affected, followed by the extravasation of blood components into the brain and compromise of normal neuronal function (Yang and Rosenberg, 2011; Jiang et al., 2018). Early BBB damage is a critical cause of parenchymal injury after stroke (Ma et al., 2020). Therefore, monitoring the integrity of the blood-brain barrier can help to understand the prognosis of ischemic stroke.

Nanoparticles cross the healthy or impaired blood-brain barrier by both active and passive processes. Imaging and quantifying their transfer rate could better characterize blood-brain barrier damage. Debatisse et al. (2020) found K trans quantification with AGuIX^®^ nanoparticles can monitor early blood-brain barrier damage and treatment effects in ischemic stroke after reperfusio. Moreover, Hou et al. (2021) found BSA-MnO2 nanoparticles (BM NPs) fabricated by a facile disinfection-mimic method showed remarkable MR imaging ability, and good biocompatibility, allowing the noninvasive timely visualization of BBB permeability in the model rats of middle cerebral artery occlusion (MCAO), which is expected to be an alternative biocompatible MR contrast agent for the noninvasive BBB permeability imaging *in vivo* (Hou et al., 2021).

#### Visualization of Collateral Vessels

Vascular occlusion is the main cause of ischemic stroke, alternative routes of blood supply to the brain are achieved by collateral vessels, which can reduce the infarction area and increase the success rate of rescuing the penumbra (Bang et al., 2015; Vagal et al., 2018). Visualization of collateral vessels is of vital importance for prompt diagnosis of the current state of ischemic stroke and timely intervention. Wang T. et al. (2018) aiming at visualizing the collaterals occurring during acute ischemic stroke prepared an integrin αv β3-specific Fe_3_O_4_–Arg-Gly-Asp (RGD) nanoprobe for magnetic resonance imaging (MRI) of the collaterals. Model rats with middle cerebral artery occlusion (MCAO) are selected for imaging studies on 7.0 Tesla MRI using a susceptibility-weighted imaging sequence. They found this nanoprobe can clearly display the collateral vessels after cerebral ischemia, which is very important for the prognosis of ischemic stroke therapies in the clinic (Wang T. et al., 2018). In a recent study, Zhang Q. et al. (2021) also developed a dibodipy-based aggregation-induced emission (AIE) fluorescent probe, THPP, which can image at a high frame rate (34 frames per second) to trace the collateral circulation process.

#### Real-Time Monitoring of Neuroinflammatory Progression in Stroke

Following the acute ischemic stroke, there are plenty of inflammatory cells, including mononuclear phagocytic system (MPS) cells and lymphocytes, around the infarction area. The secondary neuroinflammation promotes further injury, resulting in apoptosis and necrosis of neurons (Braun et al., 1996; Jayaraj et al., 2019). Therefore, a reliable tool for real-time tracking of neuroinflammatory progress is highly desired for understanding the progression of ischemic stroke.

With the help of nanoparticles, MRI enables accurate monitoring of neuroinflammatory progression. Sillerud et al. (2020) used novel anti-Iba-1-targeted superparamagnetic iron-platinum (FePt) nanoparticles in conjunction with T2-weighted magnetic resonance imaging (MRI) to measure the spatiotemporal changes of the microglial/macrophage activation in the living rat brain for 4 weeks post-stroke. They found that this approach could monitor the dynamic development of active neuroinflammation during stroke progression and treatment (Sillerud et al., 2020). Jin et al. (2016) show that magnetic resonance imaging (MRI) or Xenogen imaging combined with the labeling of SPIO-Molday ION Rhodamine-B (MIRB) can be used to monitor the dynamics of CD4(+) T cells in the ischemic area after stroke. This *in vivo* imaging approach can be used for sequential monitoring of neuroinflammation after ischemic stroke (Jin et al., 2016).

**Figure 3:**
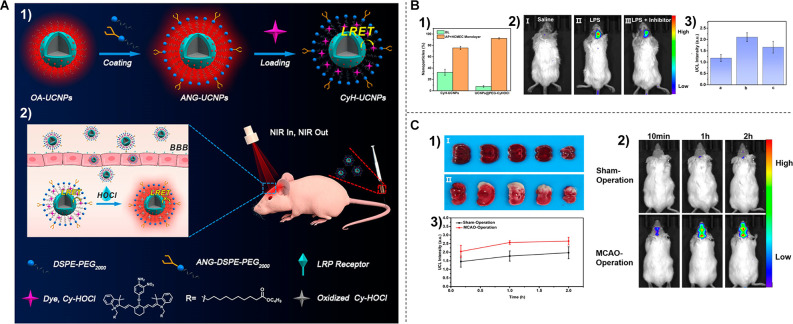
**(A)**P receptor-mediated NI endocytosis and probe response to HOCl **(2)**. **(B)** Distribution of nanoparticles after 18 h incubation in an *in vitro* blood-brain barrier model **(1)**. **(2)** *In vivo* UCL imaging of NI models after injection of saline (I), lipopolysaccharide (II), and lipopolysaccharide + minocycline (III) and their normalized UCL intensities **(3)**. **(C)** **(1)** TTC staining results of mice in sham-operated group (I) and middle cerebral artery ligation group (II). **(2)** *In vivo* UCL visualization of NI in stroke model at different time points after intravenous injection of CYH-UCNPs. **(3)** Normalized UCL intensity in **(2)**. Reprinted (adapted) with permission from Zhang et al. (2020). Copyright 2020 American Chemical Society.

Macrophages, as the most abundant inflammatory cell population in stroke lesions, can be visualized using ultrasmall superparamagnetic iron oxide (USPIO) as a cell-specific contrast agent for magnetic resonance imaging (MRI). Saleh et al. (2004) administered USPIOs to the photothrombotic cerebral infarction Wistar rat model. Through MR imaging at 7 T, the noninvasive visual monitoring of cerebral inflammation after ischemic stroke was achieved (Saleh et al., 2004). Moreover, Zhang et al. (2020) designed a blood-brain barrier (BBB) permeable and HOCl-activatable upconversion (UC) nanoprobe with NIR emission for the visual study of neuroinflammation (NI) *in vivo* (Figure 3A). Upon intravenous injection into mice, the probe crossed the BBB *via* low-density lipoprotein receptor related protein (LRP) mediated transcytosis and was then lightened up by overproduced HOCl in an NI process. This probe was able to monitor the progress of NI occurring in mice with cerebral stroke (Figures 3B,C), providing a practical tool for the noninvasive and visual assessment of NI (Zhang et al., 2020).

#### Optimization of Iodine Contrast Agent

By optimizing conventional iodinated contrast agents, nanoparticles can be used to improve ischemic stroke imaging diagnosis by acting directly as contrast agents or labeling probes to aid in the imaging diagnosis of ischemic stroke. Clinically used iodinated contrast agents (CAs) are nephrotoxic. To address this issue, Wang J. et al. (2015) synthesized a novel CT CA, polyethylene glycolated BaHoF5 nanoparticles (NPs), for CTA and CTP imaging, which can greatly improve the sensitivity and accuracy of ischemic stroke diagnosis. These drugs have unique advantages over conventional iodinated CT drugs, including metabolism primarily through the liver, lower dose requirements, and higher imaging efficiency at different voltages. Once translated, these polyglycolylated BaHoF5NPs could replace iodine-based ca for diagnostic contrast-enhanced imaging in patients with kidney disease/cardiac disease and improve overall diagnostic metrics with negligible side effects (Wang J. et al., 2015).

### Nanoparticles Enable the Diagnosis of Stroke in Body Fluids

Currently, imaging diagnosis of ischemic stroke relies on CT, MRI, and other equipment, which is not only time-consuming but also must be carried out in a qualified hospital. There is a lack of a convenient diagnostic tool to detect ischemic stroke, thereby impacting effective and efficient intervention for it at an early stage. *In vitro* diagnosis has widely been used for many diseases due to its advantages of simplicity, rapidity, noninvasiveness, and repeatability. The use of body fluid diagnostics in ischemic stroke has received increasing attention.

#### Stroke Diagnosis by Urine

The kidneys are the primary filtrating system in the human body. Which could quickly and selectively filter the biological by-products from the blood. Thus, urine represents a potential source for biomarkers in ischemic stroke (Kwong et al., 2013; Loynachan et al., 2019; Cricri et al., 2021). Thrombin is a serine protease and regulator of hemostasis that is centrally involved in the final step of the coagulation cascade and plays a critical role in the formation of obstructive blood clots, or thrombosis (Atanelishvili et al., 2014). Lin et al. (2013) design and conjugate thrombin-sensitive peptide substrates to the surface of nanoparticles to detect thrombi in living animals. Following intravenous infusion into a thromboplastin-induced mouse model of pulmonary embolism, these “synthetic biomarkers” can survey the host vasculature for coagulation and, in response to substrate cleavage by thrombin, release ligand-encoded reporters into the host urine. Their results demonstrate that synthetic biomarkers can be engineered to sense vascular diseases remotely from the urine and may allow applications in point-of-care diagnostics (Lin et al., 2013).

#### Stroke Diagnosis by Blood

For decades, because blood biomarkers have been useful, convenient, and minimally invasive in the detection and diagnosis of diseases, blood biomarkers have been used as diagnostic indicators for certain diseases (You et al., 2019; Liu et al., 2021). However, the biomarkers associated with thrombosis could not be detected directly by *in vitro* assays because the biomarkers only exist in blood clots and there are no prominent biomarkers in the blood (Su et al., 2020).

The application of nanoparticles is a good solution to this problem. Nanoparticle-based assays *in vitro* have been constructed to detect biomarkers with a low detection limit for the diagnosis of diseases at an early stage (Lei and Ju, 2012; Fu et al., 2018; Komane et al., 2020). Matrix metalloproteinases (MMPs) have been discovered to have an elevated plasma concentration in patients with ischemic stroke, especially MMP-2 and MMP-9, and they could act as a potential biomarker for the clinical diagnosis of stroke (Zhong et al., 2017; Wang C. Y. et al., 2020). Gong T et al. prepared a class of optical interference-free SERS nanotags (CO-nanotags) that can be used for multiplexed sensing of different MMPs. Such nanotags offer the advantages of convenient detection and high sensitivity. Multiplex detection with the absence of cross-talk was achieved by using CO-nanotags in an ischemia rat model, thus enabling the monitoring of ischemic stroke (Gong et al., 2017).

## Nanomaterials-Based Drug Delivery

### Limitations of Existing Thrombolytic Methods

The existing treatments for ischemic stroke mainly include pharmacological thrombolysis and mechanical retrieval, but they still have limitations in clinical application.

#### Drug Thrombolysis

Currently, treatment of ischemic stroke is limited to restoring tissue perfusion, promoting circulation, and protecting ischemic cells from death in the acute phase. Disruption of clots and restoration of blood flow using fibrinolytic drugs is the most common clinical treatment. Tissue-type fibrinogen activator (T-PA) is the only drug approved by the U.S. Food and Drug Administration (FDA) for the treatment of ischemic stroke. It promotes protein hydrolysis of fibrinogen, thereby converting inactivated fibrinogen into active fibrinogen. The fibrinolytic enzyme thus formed triggers a fibrinolytic cascade reaction that induces clot lysis by degrading the fibrin present in the clot. However, fibrinogen activator inhibitors (PAI-I, PAI-II) and fibrinolytic enzyme inhibitors (α-1-antifibrinolytic enzyme, α-2 macroglobulin) modulate circulating fibrinolytic activity (Conese et al., 1994; Chen et al., 2014), limiting the efficacy of thrombolytic therapy. In addition, fibrinolytic therapy leads to coagulation factor depletion, excess fibrin degradation products (FDPs), coagulation activation, anticoagulation, and new fibrin formation. These complex side effects can lead to many complications, mainly bleeding (Kluft et al., 2017). In addition, rt-PA has a short half-life, with an initial half-life of less than 5 min (Hemmelgarn et al., 2006), and it promotes neurodegeneration after ischemic stroke (Wang et al., 1998).

#### Mechanical Bolting

Besides administering thrombolytic drugs to the patient, another effective treatment for ischemic stroke is mechanical thrombectomy (Berkhemer et al., 2015; Campbell et al., 2015). The most popular and effective approaches for AIS thrombectomy are stent retriever techniques and contact aspiration thrombectomy techniques. The function of retrievable stents is as follows, first, the clot is crossed with a microcatheter. As the retrievable stent is unsheathed from the microcatheter, it deploys, integrating into the clot and restoring blood flow immediately by squeezing the peripheral blood vessel wall and moving the thrombus. After a short time (typically 2–4 min) of allowing the stent to integrate into the clot, the stent and microcatheter are withdrawn together with the clot (Munich et al., 2019). Compared with stent retriever techniques, contact aspiration thrombectomy may be ideally suited for distal occlusions (Muhl-Benninghaus et al., 2021). Because the microwire and microcatheter are navigable to distal vessels. The microcatheter is advanced up to or past the thrombus through a microwire, and then a large-bore aspiration catheter is advanced as close to the proximal aspect of the thrombus as possible. The large-bore aspiration catheter is connected to a source of continuous aspiration, and thrombus aspiration is attempted (Lapergue et al., 2017). Although the short-and long-term effects of mechanical thrombectomy are comparable to those of intravenous thrombolysis, it has a higher rate of systemic complications (Wang G. F. et al., 2020). And, since mechanical thrombectomy needs to be performed by a qualified neurointerventionist at comprehensive stroke centers, most hospitals remain unqualified to provide treatment. In France out of 135 nationwide neurovascular centers, only 40 are capable of performing thrombectomy to date (Forestier et al., 2020). Thus, finding innovative solutions for the treatment of ischemic stroke remains an open area of research, and nanodrugs are emerging as a promising strategy to improve the efficacy and safety of thrombolytic therapy due to their thrombotic targeting, stability, and targeted release.

### Advantages of Nanoparticles

#### Long Half-Life

Thrombolytic therapy with tissue-type fibrinogen activator (t-PA) is one of the mainstream treatments for stroke, but it has a very short half-life and requires continuous intravenous administration to maintain efficacy. This not only increases the workload of medical workers but also increases the risk of systemic bleeding in patients. Because nanomaterials can significantly prolong the half-life of drugs, they have been increasingly studied in the treatment of ischemic stroke. To broaden the therapeutic window of t-PA, Mei et al. (2019) designed t-PA-mounted, nitrogen-oxygen radical-containing, self-assembled polyionic composite nanoparticles (t-PA@iRNP). Encapsulation of t-PA in self-assembled antioxidant nanoparticles improved its bioavailability and prolonged its therapeutic window. The nanoscale formulation prevented the non-specific internalization of t-PA@iRNP in healthy cells, and this improved pharmacology significantly prolonged the *in vivo* half-life of t-PA in the body circulation. Using a mouse model of photothrombotic middle cerebral artery occlusion, they found that t-PA@iRNP treatment significantly inhibited the increase in cerebral infarct volume and improved neurological deficits after cerebral ischemia (Mei et al., 2019).

In addition, nanoparticles can also treat ischemia-reperfusion injury after ischemic stroke by prolonging the half-life of drugs with neuroprotective effects. Gallic acid (GA) is a plant polyphenol that has been used to combat ischemia-reperfusion injury (CIRI). However, the pharmacokinetic properties of GA, such as low absorption, poor bioavailability, and rapid elimination, have adversely affected its application. To enhance its effectiveness, a delivery system of ga-o-carboxymethyl chitosan nanoparticles (GA-NPs) was synthesized by Zhao et al. (2020a). GA-NPs significantly increased the area under the blood concentration-time curve and prolonged the half-life of GA. And GA-NPs had better neuroprotective effects than GA in the oxygen-glucose deprivation model and middle cerebral artery occlusion model (Zhao et al., 2020a). Acetyl-11-keto-β-boswellic acid (AKBA), the main active component of sawtooth Boswellia resin, is a new candidate for the treatment of cerebral ischemia-reperfusion (I/R) injury. However, its poor water solubility, low bioavailability, and fast clearance rate limits its efficacy. In order to enhance its potency, Ding et al. (2016) synthesized o-carboxymethyl chitosan nanoparticles (AKBA-NP) loaded with AKBA. AKBA-NPs significantly increased the area under the blood concentration-time curve and prolonged the half-life compared with AKBA. And AKBA-NPs had better neuroprotective effects in primary neurons of the oxygen-glucose deprivation (OGD) model than in animals of the middle cerebral artery occlusion (MCAO) model (Ding et al., 2016).

#### Nanomaterials Are Selective and Abundant

A large number of thrombolytic nanosystem have been developed to reduce the side effects and therapeutic costs of thrombolytic drugs, mainly including liposomal systems, polymeric nanosystems, inorganic nanosystems, and bionanosystems.

##### Liposome System

Liposomes were the first nanodrug delivery system to be successfully translated into practical clinical applications. These closed bilayer phospholipid vesicles have seen many technological advances in recent years since they were first developed in 1965 (Bulbake et al., 2017). Due to the amphiphilic nature of liposomes, they can serve as carriers for a variety of therapeutic substances. For example, hydrophilic compounds are encapsulated within an aqueous core and lipophilic compounds are dissolved within a lipid bilayer. The liposomes may comprise one lipid bilayer (monolayer liposomes) or multiple lipid bilayers (multilayer liposomes). The size distribution of liposome particles can affect the stability, encapsulation efficiency, release profile, cellular uptake of the drug, and its biodistribution (Juszkiewicz et al., 2020). Liposomes are considered one of the most promising drug delivery tools in the medical field due to their good biocompatibility and simple preparation methods, and liposome delivery changes the biodistribution of drugs and further improves the therapeutic indications of various drugs (Alves et al., 2019; Cardoso et al., 2020).

In the treatment of ischemic stroke, liposome-encapsulated fibrinogen activators (PAs) can preserve the original activity of the drug, facilitate its selective delivery and improve thrombus targeting. And with specific release at the thrombus site through membrane destabilization (including membrane fusion; Yang et al., 2016), the therapeutic potential of such liposome-based PAs has been successfully demonstrated in various *in vivo* preclinical models, and this delivery model has the advantages of good stability, low dose of thrombolytic drugs, short thrombolysis time, and few adverse effects (Koudelka et al., 2016). Vaidya et al. (2016) developed highly selective targeting-sensitive liposomes. *in vitro*, they bind to activated platelets to release streptokinase. Thrombolysis studies were performed *in vivo* on a human clot-inoculated rat model. The results showed that target-sensitive liposomes were significantly better at lysing thrombi than non-liposomal streptokinases. They also observed that target-sensitive liposomes reduced thrombus lysis time compared to streptokinase solution (Vaidya et al., 2016).

##### Polymeric Nanosystems

Polymeric nanoparticles include several types, mainly nanospheres and nanocapsule structures (Rao and Geckeler, 2011). Polymer carriers are easy to synthesize, inexpensive, biocompatible, biodegradable, non-immunogenic, non-toxic, and water-soluble. Traditionally, polymeric nanoparticles are prepared by two methods: prefabricated polymer dispersion or monomer polymerization (Rao et al., 2019). Fibrinogen activators are usually dissolved and encapsulated, or covalently attached to the surface of nanoparticles prepared from a number of polymers (Zenych et al., 2020) which can be more protected during transport in blood circulation. Bolhassani et al. (2014) describe polymeric nanoparticles as including naturally occurring hydrophilic polymers and synthetic biocompatible polymers.

##### Natural Polymers

Natural polymers such as polysaccharides (chitosan, hyaluronic acid, and alginate) and proteins (gelatin and albumin) are common. Synthetic polymers can be in pre-polymerized forms, such as polyesters like polycaprolactone (PCL), polylactic acid (PLA), or polymerized from monomers, such as polymethyl methacrylate, polycyanoacrylate (PACA), polyacrylic acid (PAA), poly(lactic acid-hydroxyacetic acid; PLGA), poly(2-oxazoline; POX), and polyamidoamines (PAMAM.) Biomacromolecule-based drug carriers are non-toxic, non-immunogenic, have high drug loading capacity, good biocompatibility, and targeting properties (Zhang Y. et al., 2018).

Polysaccharides, including fucoidan, and chitosan are one of the most widely used natural polymer carriers with the advantages of high safety, biocompatibility, and ease of preparation. Functionalized hydrogel polysaccharide sub-particles of fucoidan gum with high biocompatibility were fabricated by the inverse microemulsion/crosslinking method by Zenych et al. (2021). Fucoidan can interact with P-selectin overexpressed on activated platelets and endothelial cells in the thrombotic zone and therefore direct site-specific fibrinolysis. The thrombus-targeting properties of these particles were validated in microfluidic experiments containing recombinant P-selectin and activated platelets, at arterial and venous blood flow shear rates, and *in vivo*. Experiments on a mouse model of acute thromboembolic ischemic stroke supported the efficacy of the product, revealing a faster recanalization rate in the middle cerebral artery compared to free alteplase, resulting in reduced postischemic cerebral infarct lesions and permeability of the blood-brain barrier (Zenych et al., 2021). Polysaccharides not only act as drug carriers but also enhance the neuroprotective capacity of the carriers. Chung et al. (2018) demonstrated that adherent chitosan coating enhances the neuroprotective potential of c-phycocyanin modified liposomes (C-Pc liposomes). The application of chitosan-coated liposomes prolonged the neuroprotective time window of 6 h in a rat middle cerebral artery occlusion (MCAO) model, further improving the neuroprotective efficiency of C-Pc liposomes. And in cultured astrocytes, chitosan-coated C-Pc liposomes exhibited antioxidant activity but no cytotoxicity (Chung et al., 2018).

Among the potential natural macromolecular drug carrier systems, protein-based nanocarriers are of particular interest. Protein-based nanocarriers are promising candidates for efficient drug and gene delivery. They are capable of meeting the requirements of low cytotoxicity, abundant renewable resources, and high drug-binding capacity. In addition, their unique protein structures offer the possibility of site-specific coupling and targeting of drugs using a variety of ligands to modify the surface of protein nanocarriers (Elzoghby et al., 2012). Among them, laminin, gelatin, and albumin are the most widely used.

Ischemic stroke is caused by disruption of blood flow, resulting in focal ischemia, neuronal death, and motor, sensory, and/or cognitive dysfunction. Angiogenesis, the formation of new blood vessels from existing vessels, is necessary for tissue growth and repair. Pro-angiogenic therapy for stroke is expected to prevent excessive neuronal death and promote functional recovery. Vascular endothelial growth factor (VEGF) is a key factor in angiogenesis by promoting the proliferation, survival, and migration of endothelial cells. Oshikawa et al. developed pro-angiogenic biomaterials to support the regeneration of the injured brain. The laminin-rich (LN) sponge (LN-sponge), called porous laminin (LN), immobilizes histidine-tagged VEGF (VEGF-Histag) on it through affinity interactions. In an *in vivo* mouse stroke model, transplantation of VEGF-histag-ln sponges produced significantly greater angiogenic activity than transplantation of ln-sponges containing soluble VEGF (Oshikawa et al., 2017).

Kawata et al. (2012) developed a novel intracoronary thrombolytic smart delivery system with a strong thrombolytic effect without an increased risk of bleeding. This nanoparticle containing tissue-type fibrinogen activator (tPA), basic gelatin, and zinc ions binds to von Wilbrand factor *in vitro* and preferentially accumulates at thrombus sites in a mouse model. In a porcine model of acute myocardial infarction, plasma tPA activity after intravenous nanoparticle injection was approximately 25% of tPA and was fully recovered by transthoracic ultrasound (1.0 MHz, 1.0 W/cm^2^) (Kawata et al., 2012). Uesugi et al. (2012) designed zinc-stabilized gelatin nanocomplexes of tissue-type fibrinogen activator (t-PA) for thrombolytic therapy, in which t-PA activity could be recovered in the circulation by ultrasound irradiation. When zinc ions were added to the gelatin-t-PA complex, t-PA activity was most strongly inhibited, at 57% of the original free t-PA activity. After *in vitro* ultrasound exposure, t-PA activity was fully restored. Cell culture experiments with L929 fibroblasts showed no cytotoxicity of the complex at the concentrations used for *in vivo* experiments. The half-life of t-PA in the circulation was prolonged by complexation with gelatin and zinc ions (Uesugi et al., 2012).

Albumin is the most abundant plasma protein (35–50 g/L of human serum) with a molecular weight of 66.5 kDa. Human serum albumin (HSA) has a mean half-life of approximately 19 days. It plays an increasingly important role as a drug carrier in the clinical setting. Three main drug delivery techniques are: coupling of low molecular weight drugs to exogenous or endogenous albumin, coupling to biologically active proteins, and encapsulation of drugs into albumin nanoparticles. The first method is the most commonly used, serum albumin is capable of binding and transporting a variety of endogenous and exogenous ligands, and it can act as a reservoir for drugs, prolonging their half-life in circulation and regulating their blood concentrations (Spada et al., 2021). In their study, Liu et al. (2013) investigated the delivery efficiency of cationic bovine serum albumin-coupled tanshinone IIA polyethyleneglycolated nanoparticles (CBSA-PEG-TIIA-NPs) in the rat brain. Pharmacokinetic studies showed that CBSA-PEG-TIIA-NPs significantly prolonged the circulation time and increased the blood concentration compared to intravenous TIIA solution. Biodistribution and brain uptake studies confirmed that CBSA-PEG-TIIA-NPs had better brain administration with higher levels of drug accumulation and fluorescence quantification in the brain. CBSA-PEG-TIIA-NPs were effective in reducing infarct volume, neurological dysfunction, neutrophil infiltration, and neuronal apoptosis. In addition, it can also regulate neuronal signaling pathways. Thus, they found that CBSA-PEG-TIIA-NPs had a significant neuroprotective effect against ischemic stroke (Liu et al., 2013).

##### Synthetic Polymers

Compared to natural polymers, synthetic polymers have the advantages of high purity, good reproducibility, and ensuring a long release time of therapeutic agents (Raţă et al., 2014). They are widely used in the treatment of ischemic stroke. s-Nitrosoglutathione (GSNO) is a short-lived cerebroprotective agent that may contribute to the repair of ischemic stroke if given early for sustained administration while avoiding large reductions in blood pressure. Parent et al. (2015) developed *in situ* implants (biocompatible biodegradable copolymers) and particles (the same polymer and solvent emulsified with an external oil phase) to prolong its effect. By subcutaneous injection in Wistar rats, the particles significantly reduced brain infarct and edema volumes and were protective against stroke consequences (Parent et al., 2015). Juenet et al. (2018) designed polysaccharide polyisobutyl cyanoacrylate nanoparticles that were functionalized with fucoidan and loaded with rt-PA. They found that this nanoparticle had fibrinolytic activity *in vitro* and was bound to recombinant p-selectin and activated platelet aggregates in the mobile state. The thrombolytic efficiency was demonstrated in a mouse model of venous thrombosis by monitoring platelet density by *in vivo* microscopy. Their work established a proof of concept for the use of fucoidan-based carriers for targeted thrombolysis (Juenet et al., 2018). To alleviate the hemorrhagic side effects of thrombolytic therapy, Pan et al. (2017) developed a thrombus-targeted delivery system based on the specific affinity of Annexin V for phosphatidylserine exposed on the surface of activated platelet membranes. Namely, polycaprolactone-block-poly [2-(dimethylamino) ethyl methacrylate-block-poly (2-hydroxymethacrylate) (PCL-b-PDMAEMA-b-PHEMA (PCDH)] tri-block polymer, an amphiphilic, biodegradable biomaterial with a good thrombolytic ability (Pan et al., 2017).

To broaden the therapeutic window of t-PA and reduce its associated oxidative stress after reperfusion, Mei et al. (2019) designed t-PA mounted, nitrogen-oxygen radical-containing, self-assembled polyionic composite nanoparticles (t-PA@iRNP). Encapsulation of t-PA in self-assembled antioxidant nanoparticles improved its bioavailability and extended its therapeutic window. By covalently binding the low-molecular-weight nitro antioxidant 4-amino-2,2,6,6-tetramethylpiperidin-1-yloxy to the nanoparticle matrix, reactive oxygen species (ROS) in the ischemic semidark region were inhibited, thereby suppressing oxidative damage in the brain after reperfusion. Simultaneously, t-PA and nitrogen oxide radicals were confined and protected in the core of t-PA@iRNP, thus preventing their rapid metabolism and excretion out of the body for a long time after body circulation. Using a mouse model of photothrombotic middle cerebral artery occlusion, they found that t-PA@iRNP treatment significantly inhibited the increase in cerebral infarct volume and improved neurological deficits after cerebral ischemia. By eliminating excess ROS, t-PA@iRNP treatment also inhibited t-PA-induced subarachnoid hemorrhage (Mei et al., 2019). In addition, synthetic polymers can effectively cross the blood-brain barrier (Teleanu et al., 2019), and Jeong et al. (2019) synthesized a new EPO delivery system of bile acid-coated poly (lactic acid-hydroxyacetic acid; PLGA) nanoparticles loaded with EPO (EPO-ca-NPs), which can effectively penetrate the blood-brain barrier to compare the therapeutic effects of EPO-ca-NPs on animal models of stroke. A rat stroke model was established using the middle carotid artery occlusion reperfusion (MCAO/R) technique. The results showed that EPO-ca-NPsreduced infarct size and apoptosis more than EPO alone on postoperative day 1. In addition, EPO-ca-NPs performed better than EPO alone in terms of sensorimotor function at POD 1, 3, 5, and 7 (Jeong et al., 2019).

The biological basis for the improvement of EPO prognosis in ischemic stroke is the sustained expression of EPO and its receptor (EPO-R) in the CNS, its involvement in neuronal proliferation, migration, differentiation, and synaptic transduction, and the enrichment of stem cell nests. More importantly, recent reports on the possible effects of EPO on CNS pathological states suggest that EPO exerts neuroprotective effects by promoting antioxidant enzyme defense systems, counteracting glutamate-induced excitotoxicity, scavenging free radicals, normalizing cerebral blood flow, attenuating apoptosis and inflammation, and stimulating angiogenesis. However, the clinical application of EPO in the treatment of stroke still has limitations because it is a large glycosylated molecule with a limited ability to cross the blood-brain barrier. To overcome this major obstacle, Jeong et al. (2019) designed PLGA-NPs (EPONPs) loaded with EPO that could effectively cross the blood-brain barrier, thus improving the bioavailability of EPO. In addition, to maximize the permeability of the blood-brain barrier, they coated the surface of EPO-NPs with bile acids (CA), which are the main components of bile acids. Bile acids, including CA, are amphiphilic steroids that have been extensively studied as permeability enhancers for various biological membranes. Previous studies have shown that bile acids can cross the blood-brain barrier and induce reversible blood-brain barrier opening. These effects are thought to arise through tight junction modification, cell lysis, or receptor-mediated bile acid admixture into the lipid bilayer of endothelial cells. Together with the BBB permeability of bile acids, their detergent and hydrophobicity make bile acids widely used as encapsulants or stabilizers in the field of polymer nanotechnology (Jeong et al., 2019).

##### Inorganic Nanosystems

**Figure 4:**
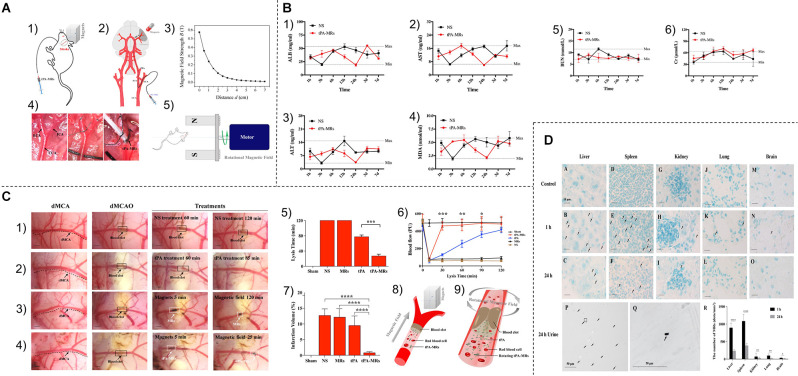
**(A)** **(1,2)** Schematic diagram of tPA-MRS injection and surgical operation. **(3)** Magnetic field strength of the magnet used for MRS or tPA-MRS guidance at different distances. **(4)** ICA injection route in the dMCAO mouse model. **(5)** During thrombolysis, the mouse head is placed in an applied eight rotating magnetic field. cCA,common carotid artery; ECA, external carotid 9 artery; ICA, internal carotid artery; PPA, pterygopalatine artery; MCA, middle cerebral artery. **(B)** tPA-MRS-mediated thrombolysis in a mouse model of stroke. **(1)** Representative images of thrombolysis in saline (NS)-treated dMCAO mice. **(2)** Thrombolysis in the tPA group within 85 min after injection. **(3)** Representative images of thrombolysis in dMCAO mice treated with MRS under four rotating magnetic fields. **(4)** The tPA-MRS solution group lysed the thrombus within 25 min under the rotating magnetic field. **(5)** Recanalization time graph of dMCAO mouse model under rotating magnetic field. **(6)** Laser Doppler detection of mice, DMCA blood flow in mice before and after thrombus formation in the DMCA and during thrombolysis. **(7)** Measurement of ischemic cerebral infarct volume at 24 h after stroke by TTC staining. **(8)** A protocol on how magnetic tPA-MRS targets on-site cerebral blood clots. **(9)** Mechanisms of magnetic tPA-MR-mediated thrombolysis. **(C)** Quantification of blood biomarkers of liver and kidney function. Mice were injected with tPA-MRs suspension or saline in the jugular vein (control group). The concentrations of three biomarkers of the liver **(1–3)**, kidney **(5,6)** function, and lipid peroxidation **(4)** were measured at different time points after injection, respectively. The data showed no significant differences between the NS group and the four tPA-MRS-treated groups for each biomarker throughout the experimental period. **(D)** Distribution and clearance of tPA-MRS *in vivo*. Liver **(A–C)**, spleen **(D–F)**, kidney **(G–I)**, lung **(J–L)**, and brain (M-30) of CD-1 mice after administration of tPA-MRS (1 mg/kg) for 1 h and 24 h. Typical Methyl Green 2 stained histological images, scale bar **(A–L)** = 4 10 μm, black arrows indicate tPA-MRS. Representative images are available for urinalysis 2 h, 4 h, 5 h after tPA-MRS **(P,Q)** injection. Black arrows indicate detection of tPA-MRS in urine and white seven arrows indicate feed residues in the metabolic cage during urine collection. **(R)** Quantitative analysis of 1 h and 24 h tPA-MRS distribution. *P < 0.05; **P < 0.01; ***P < 0.001; ****P < 0.0001. Reprinted (adapted) with permission from Hu et al. (2018). Copyright 2018 American Chemical Society.

Inorganic nanosystems mainly include magnetic nanoparticles and gold nanoparticles. Magnetic nanoparticles (MNP) have the advantages of large specific surface area, small particle size, strong superparamagnetism, low toxicity, good biocompatibility, and can be detected by MRI (Zhou et al., 2011; Mokriani et al., 2021). Initially, it was applied in the field of imaging (Starmans et al., 2013). Nowadays, MNP is also used for the slow release of thrombolytic drugs. Kempe et al. demonstrated through mathematical modeling, *in vitro* experiments, and *in vivo* experiments in rat carotid arteries, that implantation of assisted-targeting magnetic particles under the action of a magnetic field is a feasible method for local drug delivery (Kempe et al., 2010; Hu et al., 2018; Figure 4).

Gold nanoparticles (AuNPs) are commonly used materials in nanomedicine (Hsieh et al., 2012). Their unique biological properties, including antioxidant activity and drug release potential, make them promising for biomedical applications (Spivak et al., 2013). The presence of AuNPs, thiol, and amine groups allows for the coupling of various functional groups, such as targeted thrombolytic drugs or antibody products. Colloidal gold has been shown to have localized plasma surface resonance (LPSR), where gold nanoparticles can absorb light at specific wavelengths, resulting in photoacoustic and photothermal properties, making it potentially useful for high-temperature disease treatment, and medical imaging applications (Vines et al., 2019). Wang et al. (2017) prepared a NIR-triggered controlled release system using gold mesoporous silica core-shell nanospheres (Au@MSNs) and the phase change material 1-tetradecanol, formulated to release urokinase fibrinogen activator (uPA) on demand. The prepared system had a strong uPA release capacity due to the Au@MSNs-mediated photothermal effects leading to an increase in temperature. In a mouse tail thrombus model, local thermotherapy was shown to have an effective thrombolytic enhancement. Thus, based on the results, the prepared system showed potential advantages in the following aspects: control of uPA release and reduced risk of side effects, and local heat to promote thrombolysis and reduce drug dosage (Wang et al., 2017).

##### Bionic Nanoparticle System

**Figure 5:**
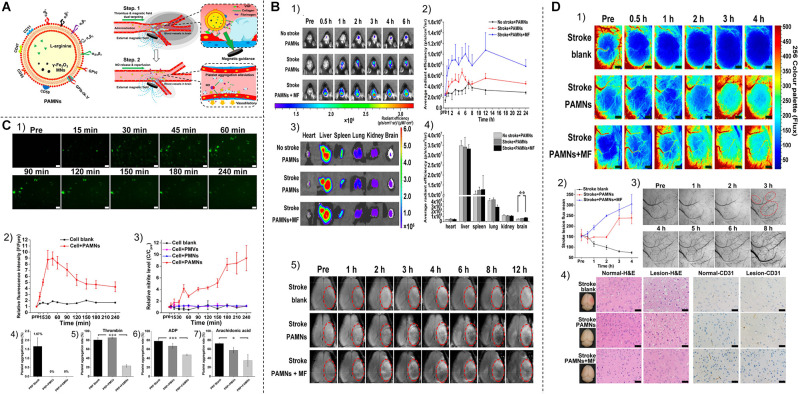
**(A)**Schematic structure and *in vivo* targeting pattern of PAMN. **(B)** NIR fluorescence imaging of PAMN *in vivo* in mice with ischemic stroke. **(1)** NIR fluorescence images of different groups of mice before and after injection of DIR-labeled PAMN. **(2)** Quantification of the mean intensity of DIR fluorescence signals in the brains of different groups of mice before and after the injection of DIR-labeled PAMN. **(3)** *In vitro* NIR fluorescence imaging was performed on the major organs resected 6 h after PAMN injection. **(4)** Quantify the intensity of DIR fluorescence signal in different groups of mice organs. **(5)** *In vivo* T2 MRI was performed before and after saline and PAMN injection. **(C)** *In vitro* characterization of nitric oxide production and biological functions. **(1)** Micrographs of NO production over time in DAF-FM DA-stained bEnd.3 cells. **(2)** Relative fluorescence intensity of NO production in bEnd.3 cells. **(3)** Relative nitrite levels in the culture supernatant were measured by Griess reaction. **(4)** PLT aggregation experiments after the addition of PMVs and PAMN to citrate-stabilized PRP. **(5–7)** Detection of thrombin, adenosine diphosphate, and AA-induced platelet aggregation rates after addition of PMV and PAMN. **(D)** Stroke reperfusion and immunohistochemical analysis. **(1)** Color laser scatter images show reperfusion of blood flow within the ischemic lesion within 4 h after thrombosis, which is comparable to the accepted treatment time window (4.5 h). **(2)** Quantitative analysis of blood flow within the ischemic lesion. **(3)** After PAMNS injection, bright-field images of the stroke vascular network were obtained at 560 nm with a multimodal optical imaging system, followed by the application of MF. **(4)** Normal brain tissue (left) and ischemic injury tissue (right) were stained for H&E and CD31. *P < 0.05; **P < 0.01; ***P < 0.001. Reprinted (adapted) with permission from Li et al. (2020). Copyright 2020 American Chemical Society.

Biomimetic nanoparticle systems combine the inherent characteristics of biological membranes with the delivery capabilities of synthetic carriers. By mimicking these biological entities such as viruses, exosomes, platelets, erythrocytes, and leukocytes, human endogenous cell-derived biomimetic drug carriers offer higher biosafety and targeting capabilities than artificial carriers (Parodi et al., 2017). Platelets play a key role in thrombosis (Kong et al., 2018). Natural platelets (PLT) can target adhesion to damaged vessels during thrombosis, so Li et al. (2018) fabricated a biomimetic nanocarrier containing a PLT membrane envelope containing L-arginine and γ-Fe_2_O_3_ magnetic nanoparticles (PAMN) for thrombus-targeted L-arginine delivery and *in situ* production of nitric oxide (NO; Figure 5A). Li et al. (2020) demonstrate that PAMNs can rapidly target stroke lesions (Figure 5B) as well as generate NO (Figure 5C) *in situ* to promote vasodilation, blood flow restoration, and stroke microvascular reperfusion (Figure 5D; Li et al., 2020). The thrombus formation site also has a large number of erythrocyte aggregates (Clemons Bankston and Al-Horani, 2019), and thus erythrocyte membranes are also widely used to target thrombotic carriers (Hill et al., 2018).

Virus-like particles (VLPs) are nanoscale biological structures composed of viral proteins whose morphology mimics that of natural viral particles but do not contain viral genetic material. The possibility of chemically and genetically modifying the proteins contained in VLPs makes them an attractive system for a variety of applications. Pitek et al. (2017) successfully applied tobacco mosaic virus (TMV) to targeted thrombolytic therapy.

Ferritin, a major iron storage protein with a hollow inner cavity (Li et al., 2009), has recently been reported to play many important roles in biomedical and bioengineering applications. Due to their unique structural and surface properties, ferritin nanoparticles can be genetically or chemically modified to impart function to their surface, and therapeutic agents or probes can be encapsulated within them by controlled and reversible assembly and disassembly. The application of functional ferritin nanoparticles in nanomedicine has attracted great interest (Wang et al., 2016). Seo et al. (2018) developed a fibrinolytic enzyme-based thrombolytic nanocage that effectively lyses clots without causing systemic fibrinolysis or disrupting hemostatic clots.

Besides, perfluorocarbon (PFC)-loaded nanoparticles (NPs) have emerged recently as powerful theranostic agents. Due to their ability to carry oxygen, PFC-loaded NPs find application in the treatment of stroke (Hoogendijk et al., 2020).

#### Targeted Release: Specific Thrombus Dissolution

Thrombus-responsive delivery systems can significantly reduce the side effects of thrombolytic agents compared to direct delivery systems. Responsive delivery systems should also incorporate a high accumulation of nanodrugs in thrombotic tissues. A responsive drug release strategy may simultaneously alter the microenvironment, resulting in a combined therapeutic effect. Although thrombus-responsive drug delivery systems are more complex than direct drug delivery systems, their enhanced biosafety provides more competitiveness for clinical applications.

##### Targeted

The greatest side effect of thrombolytic drugs is the risk of bleeding, including bleeding from the skin and mucous membranes, bleeding from the gastrointestinal tract, bleeding from internal organs, and most seriously, bleeding from the brain, which can pose a serious threat to the patient’s life. The thrombus-targeting property of nano drugs can significantly reduce this side effect (McCarthy et al., 2012), and reduce the dosage of thrombolytic drugs, which can reduce the medical burden for patients (Table 1).

**Table 1:** Methods for targeting thrombus.

Categories	Material	The strategy to target thrombus	Advantages
Targeting thrombus	cRGD peptide	GPllb/Illa complex	Higher binding affinity
			Specific binding thrombus
	CREKA peptide	Fibrin	Be linear
			Contains only five amino acid residues
	CLT peptide	Fibrin-fibronectin complexes	Specific binding thrombus
Targeting thrombus by additional conditions	Magnet	External magnetic field	Significant targeting ability
			Accelerate thrombus lysis
	Light	Local thermal gradient	Reversible
			Wireless
			On-demand remote control
	Ultrasound	Ultrasound	Specific binding thrombus

##### Targeting the Thrombus Microenvironment

**Figure 6:**
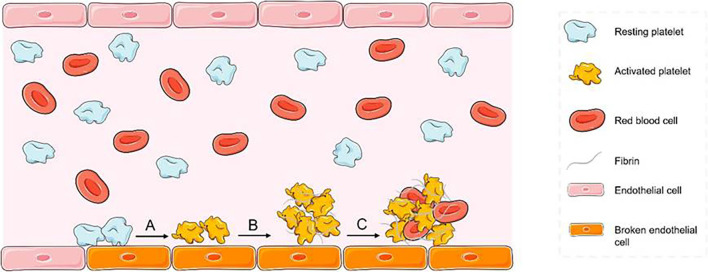
Mechanism of thrombosis diagram (A) platelets adhere to the exposed collagen surface after endothelial injury and are activated by collagen. (B) After aggregation of activated platelets, they are tightly intertwined with fibrin. (C) The initially formed thrombus nets the red blood cells and other substances in the blood.

There are multiple substances involved in the process of thrombus initiation and formation (Figure 6), and peptides that specifically bind these substances can provide thrombus targeting to nanomaterials.

***cRGD Peptide***. Activated platelets are one of the main components that promote thrombosis (Estevez et al., 2015). Platelet activation will result in the formation of a GPIIb/IIIa complex through the calcium-dependent binding of GPIIb to GPIIa, which is recognized by arginine-glycine-aspartate polypeptide (RGD) in fibrinogen. The cyclic arginine-glycine-aspartate polypeptide (cRGD) shows a higher binding affinity to the GPIIb/IIIa complex than the linear peptide (Huang et al., 2008; Zhang N. et al., 2018). Thus, the cRGD peptide is a promising thrombus-targeting peptide. Using the cRGD peptide, Chen et al. (2019) designed a new non-viral gene delivery system using the cRGD peptide for successful thrombus-targeted therapy. Similarly, Zhou et al. (2014) prepared nanoparticles with the dual function of early detection of thrombus and dynamic monitoring of thrombolytic efficiency by MRI by exploiting the property of the cRGD to specifically aggregate at the edge of thrombus.

***CREKA Peptide***. Fibrin is also one of the main components in the thrombosis process. CREKA (Cys-Arg-Glu-Lys-Ala) peptide is a thrombus-binding peptide that shows good targeting ability to fibrin. It is an ideal targeting ligand because it is linear and contains only five amino acid residues. It can assist in the massive aggregation of thrombolytic drugs at the thrombus site, thus enhancing the therapeutic effect. Kang et al. (2017) successfully fabricated nanomaterials specifically for the treatment of obstructive thrombosis using the CREKA peptide. Zhao et al. (2020b) significantly enhanced the antithrombotic activity of the drug using the CREKA peptide.

***CLT***. CLT is also a potent thrombus-targeting peptide. Seo et al. (2018) constructed a two-compartment short-length ferritin (sFt) structure with an n-terminal region fused to a multivalent clot-targeting peptide (CLT: CNAGESSKNC) and a c-terminal fusion to microfibrin (μPn); CLT was able to recognize fibrin-fibronectin complexes in thrombi, enabling efficient targeting of thrombus rupture.

##### Additional Conditions Provide Thrombotic Targeting

In addition to using peptides that specifically bind thrombotic components, additional conditions can also provide thrombotic targeting of nanomaterials.

***Magnet***. Currently, there are many nanomaterials with magnetic properties, mainly including C-type bipolar permanent magnets and superparamagnetic iron oxide. Grayston and colleagues developed superparamagnetic iron oxide nanoparticles that may be magnetically targeted for use in ischemic stroke treatment. Under the control of a magnetic device, the particles can target the ischemic cortex (Grayston et al., 2022). In the study by Huang L. et al. (2019), rtPA was covalently bonded to magnetic nanoparticles (MNP) and held at the target location by an external magnet. In a mouse model of cerebral embolism, targeting MNP-rtPA accelerated thrombus lysis and reduced infarct size (Huang L. et al., 2019). Wang et al. used a self-built C-type bipolar permanent magnet for magnetic targeting by generating a high-gradient magnetic field within a small target area. In *in vitro* experiments, nanoparticles had high retention rates in magnetic target zones with different flow rates (Wang F. et al., 2018). Thus, the magnetic targeting of nanoparticles has promising applications.

***Light***. Light as an external stimulus has the advantage of reversible, wireless, and on-demand remote control to drive the movement of micro-nanomotors (MNMs) with good spatial and temporal resolution (Xu L. et al., 2017). Therefore, the application of light-driven micromotors or nanomotors in stroke therapy holds good promise. Shao et al. (2018) reported the construction of erythrocyte membrane-encapsulated Janus polymer motors (EM-JPMs). Due to the asymmetric distribution of Au in the nanoparticles, the JPMs can move by auto thermophoretic effect under the local thermal gradient generated by near-infrared radiation. The incident intensity of the NIR laser can easily modulate the reversible “on/off” motion of the JPMs and their kinematic behavior. Therefore, it can be successfully applied to thrombus ablation (Shao et al., 2018).

***Ultrasound***. Ultrasound-targeted microbubble destruction (UTMD) has been shown to be a promising tool for delivering proteins to selected body sites. Rodriguez-Frutos et al. (2016) found that UTMD was able to deliver brain-derived neurotrophic factor (BDNF) to the brain to promote recovery of brain function and white matter. Targeted ultrasound administration of BDNF improved functional recovery associated with restoration of fiber tract connectivity and increased oligodendrocyte markers and myelin regeneration (Rodriguez-Frutos et al., 2016).

##### Multiple Targeting Strategies

In addition, two methods of targeting thrombus can also be applied simultaneously to enhance targeting ability. Because of the short half-life of recombinant tissue-type fibrinogen activator (rtPA), high-dose intravenous infusions are usually required to maintain effective drug concentrations and therefore carry a risk of bleeding. Chen et al. (2020) envisioned a dual-targeting rtPA delivery strategy that would minimize the dose required for rtPA therapy. They prepared peptide/rtPA-coupled PMNPs (pPMNP-rtPA) by co-immobilizing rtPA and RGD peptides on poly (lactic acid-glycolic acid; PLGA) magnetic nanoparticles (PMNP). pPMNP-rtPA could target thrombi through magnetic guidance and fibrin binding effects, thus showing better thrombolytic effects (Chen et al., 2020).

##### Thrombotic Microenvironment Responsiveness

There are many specific alterations in the thrombotic microenvironment, such as decreased PH, increased reactive oxygen species content, and thrombin activation. The use of these alterations allows for the specific release of the drug at the thrombus site.

***pH***. In some thrombotic tissues, such as ischemic brain tissue, the microenvironment becomes weakly acidic due to anerobic glycolysis. pH-triggered drug release is also a selective thrombolytic therapeutic strategy that does not affect normal tissues. A pH-triggered drug delivery system for thrombolytic agents was synthesized by Li et al. (2019). The release of uPA at the thrombus site is triggered by endogenous low pH to improve thrombolytic efficacy and reduce the risk of acute bleeding complications (Li et al., 2019). In addition, Cui et al. (2016) synthesized pH-sensitive polyethylene glycol-coupled urokinase nanogels (peg-ukks), which were previously reported to be a novel UK nanogel that releases the UK at a certain pH. Stromal cell-derived factor-1a (SDF-1a) is a chemoattractant molecule that plays a key role in the recruitment of endothelial progenitor cells (EPCs) to the infarct zone after stroke. Increased sdf-1 expression leads to the homing of EPCs in the infarct zone and induces neurogenesis, angiogenesis, neuroprotection, and homing of stem cells. Kim et al. (2015) use pH-sensitive micelles to efficiently deliver SDF-1a to the ischemic zone which can effectively modify the microenvironment to increase innate neural recovery processes.

***Reactive Oxygen Species, H_2_O_2_***. The thrombotic site due to ischemic stress leads to increased production of reactive oxygen species and its by-product hydrogen peroxide (H_2_O_2_; Guzy et al., 2005), so Zhao et al. (2020b) designed an H_2_O_2_-responsive nanocarrier for thrombus-targeted delivery of an antithrombotic drug (i.e., tirofiban). The nanocarrier consisted of a drug-coupled dextran nanonucleus and an erythrocyte membrane shell with a surface functionalized by the fibronectin-targeting peptide CREKA. Tirofiban is attached to dextrose *via* an H_2_O_2_ cleavable phenylboronic ester. Fibronectin-targeted erythrocyte membrane-encapsulated dextrose anhydride-tirofiban conjugated nanoparticles (T-RBC-DTC nanoparticles) scavenge H_2_O_2_ and provide a controlled release of tirofiban for site-specific antithrombotic effects. Therefore, RBC-DTC nanoparticles not only protect cells from H_2_O_2_-induced cytotoxicity but also have significantly enhanced antithrombotic activity compared to free drugs (Zhao et al., 2020b).

***Thrombin***. The specificity of thrombin release, a key event in thrombosis, means that thrombin release can act as a specific trigger for the thrombotic response delivery system (Gunawan et al., 2015). In the current study, Li et al. (2017) reported a thrombus-responsive surface coating with the ability to lyse fibrin. The coating consisted of nanocapsules (NCs) in which the fibrinolytic activator t-PA was encapsulated in a thrombin-degradable hydrogel shell. The t-PA NCs were covalently bound to a variety of materials *via* a polydopamine adhesive layer. The generated surface is treated with the antifouling agent glutathione (GSH) to prevent further interaction with blood/plasma components. t-PA NCs/gsh-coated surfaces remain stable and inert in the normal plasma environment while releasing t-PA and promoting fibrinolysis in the presence of thrombin. Fibrinolytic activity increases with increasing prothrombin concentration (Li et al., 2017).

***Activated Platelets***. Thrombosis is an important physiological process that prevents excessive blood loss. Platelets are a central component of thrombosis, and platelet activation and aggregation are key steps in thrombosis (Xu Z. et al., 2017). Therefore, activation of platelets can also be a specific trigger for therapeutic agents in ischemic stroke (Sandercock et al., 2014). Huang et al. reported a multifunctional liposome system in which tPA-loaded liposomes were polyethylene glycolized to enhance their stability and coated with conformationally restricted cyclic arginine-glycine-aspartate (CRGD) on the surface to achieve highly selective binding to activated platelets (Huang Y. et al., 2019). Activated platelets can lead to membrane fusion of liposomes in this system, and therefore tPA release can be controlled by altering the concentration of activated platelets (Koudelka et al., 2016).

##### External Conditions Promote Thrombolysis

In addition to thrombotic microenvironment responsiveness, giving external conditions (e.g., ultrasound, magnetism, light, etc.) to facilitate thrombolytic drug release from nanoparticles has also received a lot of attention.

***Ultrasound***. Ultrasound thrombolysis is a method of ultrasound-enhanced thrombolysis that has a wide range of clinical applications. Shekhar et al. (2017) designed echoliposomes loaded with recombinant tissue-type fibrinogen activator (rt-PA) for the treatment of ischemic stroke. These nanoparticles were designed to co-encapsulate cavitation nuclei to promote bubble activity upon ultrasound exposure and enable local delivery of thrombolysis. Stable cavitation improves thrombolysis by enhancing fluid mixing. Under 120 kHz intermittent ultrasound exposure, echoliposomes encapsulating inflatable microbubbles had a thrombolytic effect equivalent to that of rt-PA alone (Shekhar et al., 2017). Similarly, Bader et al. (2015) designed echoliposomes (ELIP) that encapsulate recombinant tissue-type fibrinogen activator (rt-PA) and microbubbles to improve the treatment of thromboembolic disease. In addition to the application of thrombolytic agents, the use of ultrasound contrast agents can further reduce the recanalization time of occluded vessels and improve patient prognosis. Brussler et al. (2018) investigated the effect of ultrasound thrombolysis with a new nano-ultrasound contrast agent (NUSCA). This new contrast agent is less than 100 nm in size and therefore should be able to penetrate the thrombus and achieve thrombolysis from the inside out. The experimental results show that NUSCA can induce large pores on the surface of the thrombus, leading to significant changes in the fibrin structure and thus effective lysis of the thrombus (Brussler et al., 2018).

***External Magnetic Field-Response***. External magnetic field-responsive nanoparticles are currently receiving increasing attention. Hu et al. (2018) developed a new material combining tPA with porous magnetic iron oxide (Fe_3_O_4_) microrods (tPA-mrs) for targeted thrombolysis in ischemic stroke due to distal middle cerebral artery occlusion. They found that intra-arterial injection of tPA-mrs could target cerebral blood clots *in vivo*, guided by an external magnet, and tPA was subsequently released at the embolization site. When an external rotating magnetic field was applied, the rotating tPA-mrs not only significantly improved mass transport in response to tPA-clot but also mechanically disrupted the clot network, thereby increasing clot interactions and tPA penetration (Hu et al., 2018). In addition, Cheng et al. (2014) used rotating magnetic nanomotors to enhance the mass transport of t-PA molecules at the blood clot interface for local ischemic stroke treatment. These nanoparticles could also alleviate serious side effects such as bleeding during stroke treatment (Cheng et al., 2014).

***Near-Infrared-Triggered***. Wang et al. (2017) developed a near-infrared-triggered controlled-release system consisting of gold@mesoporous silica core-shell nanospheres (Au@MSNs) formulated with the phase change material 1-tetradecanol to release urokinase plasminogen activator (uPA) on demand. au@MSNs are temperature-responsive, and the temperature increase produced by the photothermal effect allows the system to release uPA. In *in vitro* and *in vivo* experiments, local thermal therapy was validated as having an effective enhancement of thrombolysis. Thus, based on the results of the study, the system fabricated has two potential advantages: control of uPA release, thereby reducing the risk of drug side effects; and enhancement of local thrombolysis by thermotherapy to reduce drug dosage. Aided by the photothermal effect, the system showed high efficiency and on-demand drug release (Wang et al., 2017).

#### Biocompatibility and Biodegradability

Currently, nanoparticles used for ischemic stroke therapy are biocompatible and biodegradable. Biocompatibility is a fundamental requirement for biomaterials (Gabor et al., 2020). Biocompatible materials exhibit an appropriate host response (i.e., minimal disruption of normal body function) for a given application. That is, the material does not cause toxic, thrombotic, or allergic inflammatory reactions when applied *in vivo*. Two key factors determine the biocompatibility of material: the host response induced by the material and the degradation of the material in the organism’s environment. Usually, both factors should be considered (Eliaz, 2019).

The degradation rate of biomaterials is another important chemical property of nanoparticles, as it allows the release of bioactive molecules contained in biomaterials and the reconstruction of neural network structures. Depending on the polymerization process, biomaterials are expressed in different ways; for example, hydrogels are usually designed for slow degradation. They help or facilitate the development of their own extracellular matrix by exogenous cells. However, the higher their biodegradation rate, the greater the likelihood of rejection reactions. Therefore, a balance needs to be found between degradation rate and functionality (Wang et al., 2017).

#### Easily Crosses the Blood-Brain Barrier

The development of suitable drug carriers is important for improving the therapeutic efficiency of biomedical applications. Recent advances in the field of nanotechnology have paved the way for the preparation of multiple drug carriers. The treatment of ischemic stroke is not very effective due to the presence of the blood-brain barrier, which results in little penetration of the drug into the brain. Therefore, formulated nanoparticles should have the ability to cross the blood-brain barrier (BBB) for the treatment of ischemic stroke.

Lu et al. (2021) synthesized l-myostatin (LMNP) complex PLGA-functionalized magnetic fe3o4 nanoparticles (MNP) loaded with dexamethasone (dm@LMNP), which were shown to be an effective drug delivery platform that could cross the blood-brain barrier to treat ischemic stroke. Experimental results have shown that nano preparations loaded with l-myostatin have greatly facilitated the passage of drugs through the blood-brain barrier (Lu et al., 2021). Furthermore, Jeong et al. (2019) synthesized a new EPO delivery system, namely bile acid-coated poly (lactic acid-hydroxy acetic acid; PLGA) nanoparticles loaded with EPO (EPO-CA-NPs), with the aim of making EPO-CA-NPs effectively penetrate the blood-brain barrier. EPO-CA-NPs on animal models of stroke revealed that the newly synthesized brain-targeted EPO delivery system, by enabling EPO to enter the brain more efficiently, was more effective than EPO alone in stroke treatment (Jeong et al., 2019). In the treatment of cerebral infarction, Ginsenoside Rg1 (Rg1) has a pro-angiogenic and neuroprotective effect. However, the blood-brain barrier (BBB) limits the entry of Rg1 into brain tissue. The transferrin receptor (TfR) is overexpressed in the blood-brain barrier. Shen et al. (2019) prepared a TfR-targeted nanocarrier (PATRC) to penetrate the blood-brain barrier for the treatment of cerebral infarction. The nanoparticles could pass through the blood-brain barrier well and reduce brain infarct volume as well as promote microvascular regeneration in the infarcted area (Shen et al., 2019).

## Nanomaterial-Based Neuroprotection

Interrupted blood supply to the brain caused by ischemic stroke results in a loss of nutrients to the brain and induces rapid cell damage and death. Endovascular recanalization therapy aims to salvage injured brain tissue but is challenged by treatment time frames. Regenerative events initiated following brain damage are active for weeks following stroke (Dancause et al., 2005; Zhang et al., 2008). Therefore, neurorestorative therapies for stroke typically have a wide therapeutic window after stroke onset. To overcome the barriers of recanalization therapy, there is a mounting need for neurorestorative therapies to effectively treat ischemic stroke (Zhang and Chopp, 2009; Savitz et al., 2011).

**Figure 7:**
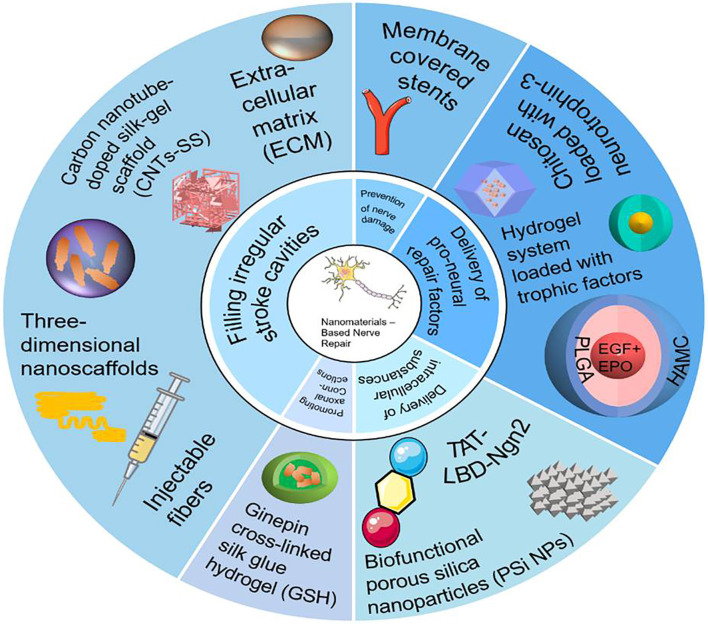
Nanomaterial-based neuroprotection approach diagram.

Neurorestorative therapies aim to amplify endogenous brain repair mechanisms and improve neurological functional outcomes after stroke by promoting neuronal plasticity, glial cell proliferation, neovascularization, angiogenesis, and arteriogenesis (Krupinski et al., 1994; Hermann and Chopp, 2012; Choi et al., 2015; Iaci et al., 2016). Non-invasive (systemic) and invasive (intracerebral) routes of administration have been preclinically and clinically explored. The main handicap of the systemic route is the inability of many biomolecules to cross a physiological barrier, the blood-brain barrier (BBB), and reach the brain with efficacy (Kinoshita, 2006; Piemontese, 2017). An alternative route for systemic administration is intracerebral. Although this route offers significant advantages, e.g., the direct administration of drugs in the area/s of interest, the complexity of this therapy makes it not directly generalized to most hospitals. Nanomaterials are a good solution to these problems (Figure 7).

### Prevention of Nerve Damage

According to the World Health Organization, stroke is “the coming epidemic of the 21st century.” Given that recent data suggests that 85% of strokes are preventable, prevention strategies are increasingly at the forefront of stroke management (Sarikaya et al., 2015). Kabinejadian et al. (2016) designed and developed a polymer membrane for carotid stenting to prevent embolic stripping from the arterial wall and consequently ischemic stroke while maintaining collateral circulation blood flow. The new membrane-covered stents showed significantly higher embolic prevention compared with the corresponding bare nitinol stents and some earlier related designs while preserving more than 93% of the original flow in the external carotid artery (ECA). These new covered stents have potential in the treatment of carotid atherosclerotic stenosis and in the prevention of late embolic strokes (Kabinejadian et al., 2016).

### Delivery of Pro-neural Repair Factors

Harnessing the regenerative potential of endogenous neural stem cells (NSCs) and neuroblastoma cells to generate new neurons is an emerging strategy for stroke treatment. Stroke induces the proliferation of new neurons in the subventricular zone, and these immature neurons migrate from the SVZ and localize within the peri-infarct tissue. However, inflammation and the inhibitory microenvironment (e.g., lack of structural support and lack of trophic factors) after traumatic brain injury (TBI) prevent NSCs from generating new functional neurons to restore brain function (Ohab and Carmichael, 2008). To address these issues, Hao et al. (2017) reported that a biodegradable material, chitosan, loaded with neurotrophin-3 (NT3) and injected into the injury site after TBI, effectively promotes the proliferation and migration of endogenous NSCs to the injury area. NSCs differentiate and mature into functional neurons, forming nascent neural networks that further integrate into existing neural circuits and restore brain function. The nt3-chitosan in this particle has three main effects, namely promoting neurogenesis, anti-inflammation, and promoting revascularization, causing significant tissue regeneration after traumatic brain injury (Hao et al., 2017).

In addition, trophic factors released by stem cells play an important role in promoting stem cell growth, and the hydrogel system studied by George et al. (2018) can deliver these important molecules. The important trophic factors secreted by stem cells can be effectively released from the multicomponent hydrogel system into the post-stroke environment, thereby improving stroke recovery (George et al., 2018). Another promising strategy to achieve ischemic brain tissue repair is to stimulate endogenous neural stem/progenitor cell growth through sequential delivery of epidermal growth factor (EGF) and erythropoietin (EPO). Wang Y. et al. (2013) designed a novel delivery system that bypasses the blood-brain barrier and releases growth factors directly to the brain. Sequential release of both growth factors significantly induced tissue repair. To control the release of growth factors, they encapsulated pegylated EGF (EGF-peg) in poly (lactic acid-hydroxyacetic acid; PLGA) nanoparticles and EPO in biphasic particles consisting of a PLGA core and a poly (sebacic) acid (sebacic) coating. eGF-PEG and EPO polymer particles were dispersed in a hyaluronic acid methylcellulose (HAMC) hydrogel. This hydrogel spatially restricted the release of particles and attenuated the inflammatory response of brain tissue. In a mouse stroke model, their complex-mediated, sequential infusion of EGF-PEG and EPO resulted in tissue repair that minimized tissue damage compared to ICV infusion (Wang Y. et al., 2013).

### Delivery of Intracellular Substances That Promote Nerve Repair

Harnessing the regenerative potential of adult neural stem cells (NSCs) and neuroblastoma cells to generate new neurons is an emerging therapeutic strategy for ischemic stroke. Neuroblasts differentiate towards the neural stem cell lineage and have a low proliferation rate. In stroke, the proliferation of neuroblasts in the neurogenic zone increases, but the survival of neuroblasts that migrate to the ischemic zone is low. To address this problem, Deng et al. (2013) reported TAT-LBD-Ngn2 fusion proteins constructed by fusing the TAT structural domain and the LBD structural domain with neurogenin-2 (Ngn2). Among them, Ngn2 is a neurogenin that promotes the survival and differentiation of neural precursor cells and is an attractive candidate for the treatment of cerebral ischemia-reperfusion injury. A focal cerebral ischemia model in C57BL/6 mice showed that TAT-LBD-Ngn2 efficiently crossed the blood-brain barrier, aggregated in the ischemic zone, and was incorporated consistently into neurons, attenuating neuronal degeneration and apoptosis. This leads to a reduction in brain infarct volume (Deng et al., 2013). Furthermore, the growth of neuroblastoma cells can be promoted by affecting their intracellular pathways. Balasubramanian et al. (2020) designed biofunctional porous silica nanoparticles (PSi NPs) that bind to a specific antibody against polysialinized neural cell adhesion molecules (PSA-NCAM). PSi NPs loaded with the small molecule drug SC-79 were able to increase the activity of the Akt signaling pathway in dual corticosteroid-positive neuroblastoma cells in both cultured and living cells. This promotes neuroblastoma cell differentiation, maturation, and survival (Balasubramanian et al., 2020).

Neural precursor cells (NPCs) designed to express therapeutic genes may also be valuable tools for restorative cell therapy and for targeting therapeutic genes to diseased brain regions. Here, Schmidt et al. (2007) report the identification of nasopharyngeal carcinoma-specific ligands from a phage display peptide library and demonstrate their potential to selectively transfer adenovirus-mediated genes into adult mouse nasopharyngeal carcinomas. The identified peptides mediated specific binding and internalization of the virus to cultured neurospheres. Importantly, peptide-mediated infection of adenoviral vectors was restricted to pNestresen fluorescent protein transgenes or precursor cells in the hippocampal dentate gyrus of C57BL/6 mice. Their approach represents a novel way to specifically manipulate NPCs in the adult brain and may have important implications for the use of precursor cells as therapeutic vectors in the central nervous system (Schmidt et al., 2007).

### Promoting Axonal Connections

**Figure 8:**
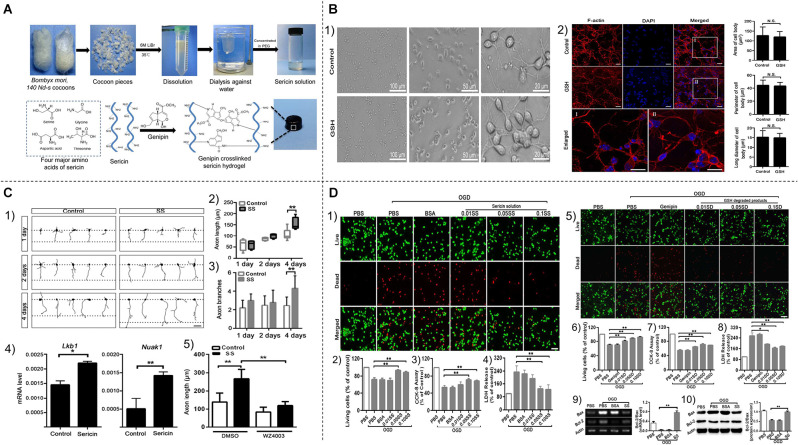
**(A)**Schematic diagram of the preparation of silk-based ginsenosidecross-linked silk glue hydrogel (GSH). **(B)** Effectiveadhesion, growth, and spreading of primary neurons on the GSHsurface. **(C)** Filament gum promotes axonal growth andbranching *in vitro*. **(1**) Axons extending fromneurons were observed after 1, 2, and 4 days of treatment with orwithout filamentous glue solution (SS). Scale bar, 40 μm.**(2 and 3; 1)** Quantification of neuronal axon length and axonbranching (*n* = 20–25). **(4; 1)** mRNA levels of Lkb1 and NUAK1 genes in primary neurons after 48 h of treatment shown in **(1)**. **(5)** Axon lengths of DMSO or WZ4003 (an Lkb1-NUAK1 pathway inhibitor) treated primary neurons in the presence (control) or absence of filamentous glue (SS). **(D)** Neuroprotective effects of filamentous gum solution and glutathione degradation products on primary cortical neurons against oxygen-glucose deprivation (OGD) injury. **(1)** Neurons were treated with PBS, BSA, and silk gum solution (SS) for 1 h, followed by routine incubation for 24 h. Neurons treated with PBS but not OGD were the non-OGD control group. All cells were stained for live and dead cells. **(2–4)** Quantification of the percentage of live cells (normalized to non-OGD control; **2**), CCK-8 assay of od values at 450 nm **(3)**, and LDH release from neurons that received the indicated treatments in **(1; 4)**. Scale bar, 50 μm. Each experiment was performed three times, per treatment; three experiments were repeated. **(5)** Neurons were treated with specific concentrations of PBS, BSA, and GSH degradation products (SD) for 1 h, followed by routine incubation for 24 h. Neurons treated with PBS but not OGD were non-OGD controls. All cells were stained for live and dead cells. **(6–8)** Percentage of live cells (normalized to non-OGD control; **6**), OD at 450 nm determined by CCK-8 **(7)**, and LDH release from neurons receiving the indicated treatments in **(5; 8)**. **(9)** The relative mRNA levels of Bcl-2 and Bax were detected by real-time fluorescent quantitative PCR (Real time PCR, left), and the band intensity ratio of Bcl-2 and Bax was quantified (right). **(10)** Protein expression of Bcl-2 and Bax in each treatment group was analyzed by Western blotting (left), and the band intensity ratios of Bcl-2 and Bax were quantified (right) accordingly. *P < 0.05; **P < 0.01; Student’s *t*-test. Reprinted (adapted) with permission from Wang et al. (2015). Copyright 2015 American Chemical Society.

Ischemic stroke brain injury leads to rapid cell death and disruption of functional circuits in the affected area. As injured tissue recovers from events associated with cell death, tissue regeneration processes are activated that can lead to some degree of functional recovery within a few months. Axonal sprouting of surviving neurons and the formation of new synapses help to re-establish some of the lost functions (Wieloch and Nikolich, 2006). Wang Z. et al. (2015) prepared a ginepin cross-linked silk glue hydrogel (GSH) with a porous structure and a mild swelling rate using the natural protein silk glue from silk (Figure 8A). In *in vitro* experiments, GSH supported the effective attachment and growth of neurons (Figure 8B). Moreover, filamentous gliadin is intrinsically neurophilic and neuroprotective, promoting axon extension and branching (Figure 8C) as well as preventing hypoxia-induced cell death in primary neurons (Figure 8D). Notably, these functions are produced by degradation products of GSH, which may obviate the need to integrate expensive cytokines (Wang Z. et al., 2015).

In addition, plant viruses have a wide range of applications in promoting neural axonogenesis. Feng et al. produced baculovirus particles displaying integrin-binding motifs that are biocompatible with the mouse neurospinal-derived cell line Neuro 2a (N2a) and promote axonal growth of N2a. By applying shear forces, transgenic viruses can be assembled in capillaries in an aligned orientation. The obtained aligned substrate is able to determine the directional protrusion growth of N2a cells. Therefore, this method has potential application in neural tissue engineering as a neural tube tract for repairing nerve damage (Feng et al., 2015).

Similarly, Wu et al. prepared electroactive nanofibers by *in situ* polymerization on the surface of tobacco mosaic virus (TMV) using polystyrene sodium sulfonate (PSS) as a dopant. These electroactive TMV/PANI/PSS nanofibers were used to support the growth of neural cells, leading to an increase in the length of neural protrusions. The TMV-based electroactive nanofibers could align in capillaries, direct the direction of neural protrusion growth, increase the proportion of cells with neural protrusions, and lead to bipolar cell morphology. Their results suggest that the electroactive and morphological cues provided by TMV/PANI/PSS nanofibers can synergistically stimulate neuronal cell differentiation and protrusion growth, which makes them a promising scaffold material for neural tissue engineering (Wu et al., 2015).

### Filling Irregular Stroke Cavities

Severe ischemic stroke damages neuronal tissue and forms irregular stroke cavities with no supporting structures. Ischemic brain injury results in a reduction in brain volume (atrophy), including a decrease in extracellular matrix (Moreau et al., 2012). This alteration is irreversible under the current treatment paradigm. However, the adult mammalian brain has endogenous neurogenesis, which is upregulated after injury and contributes to the repair of brain tissue. This endogenous repair response is necessary for tissue regeneration. However, scarring and cavitation around the core of the lesion provide unfavorable conditions for tissue regeneration in the brain. Implantation of biomaterials that provide structural and functional support is thought to facilitate the growth of regenerative neural networks to promote functional tissue reconstruction after CNS injury. Extracellular matrix scaffolds from mammalian tissues retain many bioactive molecules and have recently been recognized for their ability to repair the central nervous system (Meng et al., 2014).

Ghuman et al. (2016) used cell-free extracellular matrix (ECM), formulated as a hydrogel that could be produced *in situ* within the cavity formed by stroke, as an alternative to necrotic debris and to promote infiltration of host brain cells. This hydrogel promotes a significant acute endogenous repair response and is a promising therapeutic material for ischemic strokes (Ghuman et al., 2016). Later, they proposed implanting porcine-derived bladder matrix (UBM) extracellular matrix (ECM) hydrogel into the stroke cavity, and ECM hydrogel implantation into the stroke cavity attracted endogenous cells, this hydrogel partially induced repair of neural tissue (Ghuman et al., 2018). Wang J. et al. reported an injectable, photoluminescent, carbon nanotube-doped silk-gel scaffold (CNTs-SS) with programmable shape memory properties and the ability to pre-design its shape to precisely match any irregularly shaped cavity. By applying them to a preclinical stroke model, they found that CNTs-SS with customized shapes could recover the pre-designed shapes to fit the cavity well after successful injection into the cavity. In addition, the near-infrared photoluminescence of CNTs-SS allowed for non-invasive real-time tracking after implantation *in vivo* (Wang J. et al., 2021). The filament-based biomaterials studied by Fernandez-Garcia et al. (2018) can effectively support the survival of implanted mesenchymal stem cells (mSCs) in the brain. In addition, filamentin protein hydrogels enhance the ability of MSCs to protect against brain injury after cerebral infarction and induce delayed plasticity in the tissue cortex surrounding the injury (Fernandez-Garcia et al., 2018).

**Figure 9:**
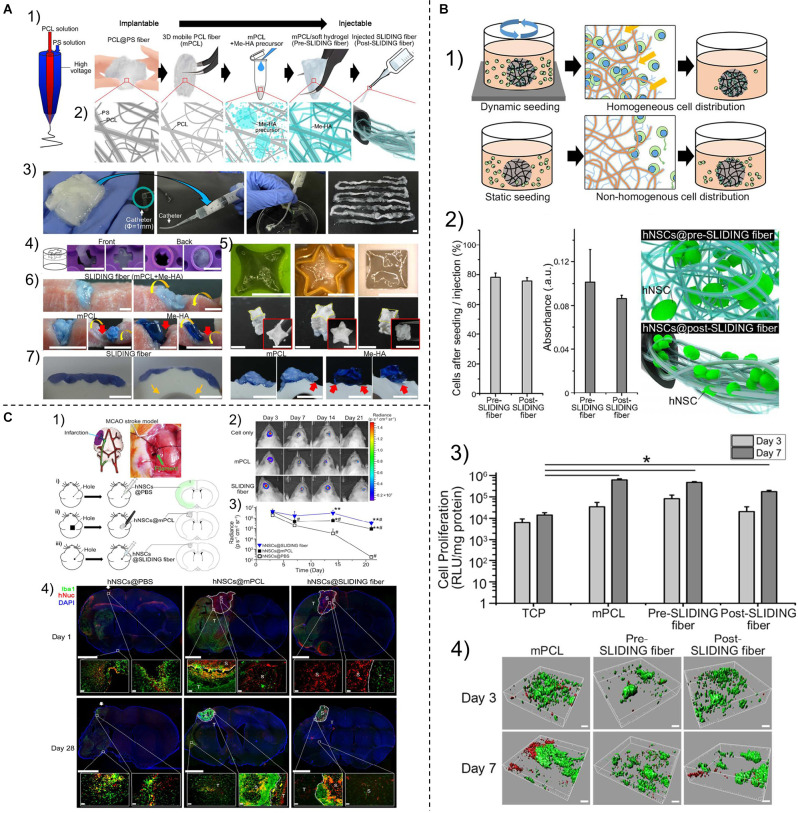
**(A)**Preparation of sliding fibers and their representative characteristics. **(B)** *In vitro* culture of human neural stem cells within sliding fibers. **(1)** Schematic diagram showing differences in cell seeding methods. **(2)** Percentage (left panel) and activity (middle panel; CCK-8) of cells remaining in fibers before and after sliding. **(3)** Cell proliferation after 3 and 7 days of culture in each system [i.e., tissue culture plate (TCP), mPCL sponge, and fibers before and after slip]. Cell proliferation was quantified by monitoring the luciferase activity of hNSCs. **(4)** CLSM images of hNSCs grown within the mPCL, before or 3 or 7 days after sliding. Live and dead hNSCs were stained with FDA (green) and PI (red), respectively, and the distribution of live/dead hNSCs in each system was reconstructed in three dimensions using IMARIS software. **(C)** Sliding fibers as hNSC carriers in rats with ischemic stroke. **(1)** Schematic diagram of the preparation of the MCAO stroke model and the route of hNSC administration into the rat brain cavity. **(2)** Images of light signals emitted by hNSCs expressing active luciferase implanted or injected in the lesion site at 3, 7, 14, and 21 days after treatment. **(3)** Emitted light intensity within the region of interest (*n* = 3) covering the injection or implantation site. **(4)** Representative images of immunohistological staining for human nucleus (HNuc; red) and microglia-specific protein Iba1 (green). *P < 0.05; **P < 0.01. Reprinted (adapted) with permission from Lee et al. (2016). Copyright 2016 American Chemical Society.

To regenerate damaged neural tissue, Boni et al. made three-dimensional nanoscaffolds (3DNSs) from a mixture of biomaterials of filamentous protein (SF), polyethylene glycol (PEG), and polyvinyl alcohol (PVA). The 3DNSs have the potential to be directly implanted into the central nervous system. *In vitro* and *in vivo* experiments showed that the particles increased cell viability in the ischemic zone and inhibited the proliferation of reactive astrocytes (Boni et al., 2020). There is also an increasing interest in designing biomaterial systems that mimic fibers, and natural extracellular matrix to enhance the effectiveness of various therapeutic tools. Lee et al. developed a smart technique for minimally invasive injection of 3D electrospun silk fibers. Combining electrospun silk fibers with lubricated hydrogels produces fiber structures called slidable, ejectable, and gel-like (sliding) fibers. These sliding fibers can pass smoothly through the catheter, filling any cavity while maintaining fiber morphology (Figure 9A). The resulting injectable fibers provide a good environment for human neural stem cells (hNSC) to proliferate and form neurospheres within the fiber structures without affecting the viability of the hNSC (Figure 9B). Sliding fibers exhibited superior cell carrier properties in an animal model of middle cerebral artery occlusion (MCAO) stroke. In this model, the application of sliding fibers prolonged the survival of administered hNSCs by blocking microglia infiltration during the early acute inflammatory phase (Figure 9C; Lee et al., 2016).

## Discussion and Conclusions

Due to the narrow therapeutic window for acute ischemic stroke, timely diagnosis and rapid cerebral blood flow restoration and/or microcirculatory remodeling are critical for stroke outcome (Merino and Warach, 2010; Lee et al., 2018). Nanomaterials have the advantages of good biocompatibility, high safety profile, and unique optical properties (Zeng et al., 2021), thus nanomaterials have a promising future as an emerging ischemic stroke diagnostic reagent. A large number of nanomaterial-based diagnostic imaging systems have been developed, including imaging nanoparticles that can specifically bind to thrombi, ischemic semidark zones, and other sites; nanoparticles that detect blood-brain barrier integrity, collateral vascular compensation, and inflammatory cell infiltration in local ischemic areas; and nanoprobes that can be used for the diagnosis of ischemic stroke body fluids. These diagnostic imaging systems can not only identify potential biomarkers and expand the scope of conventional imaging but also monitor the dynamic development of ischemic stroke, giving the possibility of early diagnosis and timely treatment of ischemic stroke. However, these diagnostic imaging systems can only detect unilateral physiological changes, which cannot meet the clinical needs. Multifunctional nano-diagnostic reagents should be constructed to provide detection of multifaceted physiological changes in ischemic stroke, leading to a more accurate diagnosis of the disease. In addition, these systems should have microenvironment-responsive linkers in the ischemic region that allow controlled drug release in the stroke region to improve imaging.

In addition to early diagnosis, ischemic stroke is primarily caused by obstruction of blood vessels supplying blood to the brain (Chang et al., 2019). Rapid recanalization of vascular obstruction is therefore also crucial. Innovative nanomedicine approaches have been widely proposed for targeted thrombolytic therapy to address the challenges of systemic drug delivery. The benefits of nanomaterials in the treatment of ischemic stroke disease include prolonged blood half-life of thrombolytic drugs, reduced doses of thrombolytic drugs used, and reduced systemic bleeding complications. Recently, a large number of nanocarriers have been extensively studied, including liposomes, polysaccharides, proteins, polymers, inorganic nanoparticles, and bionanoparticles. These nanocarriers can not only aggregate at the site of cerebral ischemia *via* fibrin or platelet-specific binding peptides, etc., but also localize the release of thrombolytic drugs in the ischemic zone for rapid thrombus dissolution. It is a promising treatment for ischemic stroke. However, the immunogenicity of bionanoparticles, the cytotoxicity of inorganic nanocarriers, and the complexity of the nanocarrier preparation process are issues that cannot be ignored. Therefore, FDA-approved biocompatible and fully biodegradable materials should be selected, as well as nanocarriers should be manufactured at scale according to good manufacturing practices (GMP). In addition, multi-targeted nanocarriers should be designed to improve the thrombolytic effect.

Loss of neuronal cytosol, axons, and associated glial cells is a neuropathological hallmark of ischemic stroke. Recently, nanomaterials have been widely used to repair damaged neural tissue, including nanoparticles loaded with neurotrophic substances such as neurotrophic factor, epidermal growth factor, or erythropoietin, nano preparation that can fill irregular cavities in ischemic areas after stroke, and implanted biomaterials that facilitate the growth of regenerative neural networks. Although strategies to repair damaged neural tissue using nanomaterials have been shown to potentially improve neurological outcomes and reduce infarct size, many questions remain. For example, the exact cellular and biochemical mechanisms by which nano scaffolds induce neural repair and whether transplanted nanomaterials produce a secretion of neurotrophic factors that stimulate endogenous repair remain unclear. And most of the nerve repair systems used are synthetic nanomaterials with poor biocompatibility. A low-cost, simple-to-synthesize, able to gel autonomously in response to applied stimuli (e.g., pH or temperature changes), and capable of modulating and supporting nerve cell function in ischemic areas should be developed. In addition, a multifunctional biomimetic strategy should be established that enables not only stimulus responsiveness but also the delivery and precise control of stem cell behavior in neural tissue regeneration applications.

Today, real-time diagnostic localization of thrombus, visualization of thrombolytic drug delivery, and combination of thrombolytic drugs with neuroprotective agents are the main trends in the use of nanomaterials for ischemic stroke therapy. Research in these areas should be accelerated to accommodate clinical needs.

## Author Contributions

NL and HT conceived the project, supervised XL’s writing and took part in edition and review. XL and NL performed the conceptualization, references reading, and writing. All authors contributed to the article and approved the submitted version.

## Conflict of Interest

The authors declare that the research was conducted in the absence of any commercial or financial relationships that could be construed as a potential conflict of interest.

## Publisher’s Note

All claims expressed in this article are solely those of the authors and do not necessarily represent those of their affiliated organizations, or those of the publisher, the editors and the reviewers. Any product that may be evaluated in this article, or claim that may be made by its manufacturer, is not guaranteed or endorsed by the publisher.
